# Combination therapy with c-met inhibitor and TRAIL enhances apoptosis in dedifferentiated liposarcoma patient-derived cells

**DOI:** 10.1186/s12885-019-5713-2

**Published:** 2019-05-24

**Authors:** Eun Byeol Jo, Young Sang Lee, Hyunjoo Lee, Jae Berm Park, Hyojun Park, Yoon-La Choi, Doopyo Hong, Sung Joo Kim

**Affiliations:** 1 Sarcoma Research Center, Samsung Biomedical Research Institute, 81 Irwon-ro, Gangnam-gu, Seoul, 06351 South Korea; 2 Samsung Advanced Institute for Health Sciences and Technology, SKKU, Seoul, Republic of Korea; 3 Personalized Medicine, Children’s Cancer Institute Australia, Sydney, NSW Australia; 4 Department of Surgery, Samsung Medical Center, SungKyunKwan University School of Medicine, 81 Irwon-ro, Gangnam-gu, Seoul, 06351 South Korea; 5 Department of Pathology, Samsung Medical Center, Seoul, Republic of Korea

**Keywords:** Human recombinant TRAIL, C-met inhibitor, Liposarcoma, DR5, C-met receptor, DR5 dependent apoptosis, Combination treatment

## Abstract

**Background:**

Liposarcoma (LPS) is a tumor derived from adipose tissue, and has the highest incidence among soft tissue sarcomas. Dedifferentiated liposarcoma (DDLPS) is a malignant tumor with poor prognosis. Recurrence and metastasis rates in LPS remain high even after chemotherapy and radiotherapy following complete resection. Therefore, the development of advanced treatment strategies for LPS is required. In the present study, we investigated the effect of tumor necrosis factor-related apoptosis-inducing ligand (TRAIL) treatment, and of combination treatment using TRAIL and a c-Met inhibitor on cell viability and apoptosis in LPS and DDLPS cell lines of tumor necrosis factor-related apoptosis-inducing ligand (TRAIL) treatment, and of combination treatment using TRAIL and a c-Met inhibitor.

**Methods:**

We analyzed cell viability after treatment with TRAIL and a c-Met inhibitor by measuring CCK8 and death receptor 5 (DR5) expression levels via fluorescence activated cell sorting (FACS) in both sarcoma cell lines and DDLPS patient-derived cells (PDCs). Moreover, we validated the effects of TRAIL alone and in combination with c-Met inhibitor on apoptosis in LPS cell lines and DDLPS PDCs via FACS.

**Results:**

Our results revealed that combination treatment with a c-Met inhibitor and human recombinant TRAIL (rhTRAIL) suppressed cell viability and induced cell death in both sarcoma cell lines and DDLPS PDCs, which showed varying sensitivities to rhTRAIL alone. Also, we confirmed that treatment with a c-Met inhibitor upregulated DR5 levels in sarcoma cell lines and DDLPS PDCs. In both TRAIL-susceptible and TRAIL-resistant cells subjected to combination treatment, promotion of apoptosis was dependent on DR5 upregulation.

**Conclusion:**

From these results, our findings validated that DR5 up-regulation caused by combination therapy with a c-Met inhibitor and rhTRAIL enhanced TRAIL sensitization and promoted apoptosis. We propose the use of this approach to overcome TRAIL resistance and serve as a novel treatment strategy for clinical trials.

**Electronic supplementary material:**

The online version of this article (10.1186/s12885-019-5713-2) contains supplementary material, which is available to authorized users.

## Background

Soft tissue sarcoma (STS) is a malignant tumor that develops in soft tissues, in addition to bone tissues. STS is classified into 50 subtypes based on its tissue of origin, pathological characteristics, clinical behavior, and metastatic potential. The most prevalent subtypes of STS include liposarcoma (LPS), undifferentiated pleomorphic sarcoma (UPS), leiomyosarcoma (LMS), and gastrointestinal stromal tissue (GIST). STSs comprise less than 1% of all cancer cases, and therapeutic studies on these cancers are usually limited to a small number of patients [1] Thus, there are also limited treatment options, except for GIST, where imatinib is used as the primary treatment [2]. Liposarcoma is the most common type of STS, and includes well-differentiated liposarcoma (WDLPS) and dedifferentiated liposarcoma (DDLPS). WDLPS and DDLPS are usually found to co-exist within a large tumor mass, but exhibit different clinical behaviors. DDLPS is more aggressive, has a higher rate of mitosis and proliferation and is therefore characterized by a high frequency of recurrence and metastasis. On the other hand, WDLPS is a benign tumor that has a low frequency of metastasis and recurrence [3]. DDLPS, which mainly develops in the retroperitoneum, is highly aggressive, and often exhibits resistance to chemotherapy and radiotherapy treatments; as a result, patients with DDLPS have a poor prognosis after surgical resection of these tumors [4]. Therefore, DDLPS remains one of the most challenging cancers to treat, and further studies are required to develop targeted therapies that can effectively overcome the drug resistance often associated with these tumors.

TNF-related apoptosis-inducing ligand (TRAIL) is a pro-apoptotic protein that exerts strong cytotoxic effects on cancer cells. TRAIL-based therapeutic treatments developed for clinical applications include recombinant TRAIL proteins, TRAIL-engineered viruses, and TRAIL-expressing engineered cells [5]. TRAIL is one of the alternative treatment drugs that is considered to have the highest therapeutic potential for the treatment of cancers with limited treatment options [6]. Although TRAIL is a promising drug for cancer therapy, the effect of TRAIL has exhibited varying sensitivities according to diverse types of sarcomas in previous studies [7–9]. Therefore, the development of therapeutic strategies that overcome TRAIL resistance is required for effective treatment of sarcoma patients. Accordingly, understanding the molecular mechanisms involved in TRAIL resistance in cancer cells and developing novel anti-cancer drugs that synergize with the various cancer cell responses to TRAIL are prerequisites for a more extensive and successful application of TRAIL-based therapies in the future [10–13].

Recent studies have reported that c-Met is highly expressed in DDLPS tissues, and a novel c-Met inhibitor which can target the activated c-Met was found to have high anticancer efficacy against DDLPS cells [14]. Many other cancers have been shown to highly express c-Met, and these receptors have been targeted for downregulation to prevent further recurrence or metastasis. Therefore, drugs that target the c-Met tyrosine kinase are often used in combination with other drugs, such as inhibitors of Wnt, EGFR or TRAIL, and it may give alternative possibilities therapeutically [15–19].

Considerable research has focused on the treatment of cancer via the targeting of TRAIL signaling. However, it is increasingly reported that there is a predominance of TRAIL resistance in many human primary carcinomas; therefore, the increased sensitization of these cells to TRAIL is required for effective induction TRAIL-based apoptosis [20]. Although sensitivity to TRAIL has been shown to be restored in TRAIL-resistant cancer cells following chemotherapy and radiation therapy [21, 22], the sensitivity of TRAIL-resistant cancer cells has also been shown to be altered through regulation of c-Met, which can lead to the eventual triggering of TRAIL-induced apoptosis. Recently, there have been two studies that have demonstrated that combination treatment with a c-Met inhibitor and TRAIL resulted in induction of apoptosis in glioma and papillary thyroid carcinomas, almost all of which were due to increased DR5 and inhibition of the activated c-Met pathway [23, 24]. Here, we introduced the use of a c-Met inhibitor to promote TRAIL sensitization in DDLPS. c-Met is a pro-oncogene whose expression levels are associated with cancer aggressiveness. Many studies have verified that overexpression or abnormal activation of c-Met leads to increased cell proliferation, migration, and invasion, as well as inhibition of apoptosis [25]. Various drugs that target c-Met signaling have been approved and are currently in the clinical stages of testing [26].

In this study, we treated PDCs isolated from liposarcoma patients with a combination of a c-Met inhibitor and TRAIL. We investigated whether this combination treatment could overcome TRAIL resistance and enhance TRAIL-induced apoptosis in DDLPS PDCs. We also explored methods that could increase sensitivity to TRAIL treatment by up-regulating expression levels of DR5 toward TRAIL-resistant cells. Here, we showed that PDCs showed varying sensitivities to TRAIL. Pre-treatment with a c-Met inhibitor, followed by treatment with TRAIL, significantly enhanced TRAIL-induced apoptosis in TRAIL-resistant DDLPS cells via up-regulation of death receptor 5 (DR5).

Our results demonstrated that combination therapy with a c-Met inhibitor and TRAIL effectively decreased the survival of patient-derived liposarcoma cells (PDCs) that demonstrated TRAIL-resistance. Also, these data provide a valuable basis for the use of a combination therapeutic strategy for the treatment of patients with DDLPS to suppress recurrence and metastasis after surgical removal of tumors, since subsequent relapse is the primary cause of mortality within 5 years of surgical resection in these patients. Furthermore, c-Met inhibitors, such as crizotinib, and TRAIL-based drugs are currently in the clinical stages of testing, and may prove to be potential medications for patients with DDLPS.

## Methods

### Cells and reagent

Human recombinant TRAIL protein (rhTRAIL) was purchased from R&D Systems (R&D Systems, Minneapolis, MN, USA). c-Met inhibitors (PHA665752: PHA; PF02341066:crizotinib: PF) were bought from Sellekchem (Houston, TX, USA). The isolation of mesenchymal stem cells (MSCs) from human tissues was performed as described previously [27]. The malignant fibrous histocytoma (MFH) cell line MFH-ino was obtained from the Rikagaku Kenkyusho Cell Bank (Kanagawa, Japan). Human liposarcoma and fibrosarcoma cell lines (SW872 and HT1080, respectively) were purchased from the Korean Cell Bank (Seoul, Republic of Korea). An established DDLPS cell line and a human liposarcoma cell line (LPS224 and LPS246, respectively), were kindly provided by Dr. Dina Lev (The University of Texas MD Anderson Cancer Center, Houston, TX, USA). DDLPS patient-derived cells (PDCs); 11GS-013(< 60 years old, Female, retroperitoneum), 11GS-079 (> 60, Male, retroperitoneum), 11GS-099 (> 60, M, retroperitoneum), 11GS-106 (> 60, F, retroperitoneum), 14GS-076(< 60, F, retroperitoneum) were isolated from the tumor tissues of patients of the Samsung Medical Center (SMC; Republic of Korea). The tumor material was excised aseptically and then processed to isolate the primary tissue, as described previously [28]. Briefly, primary surgical and core biopsy tissue samples were transported from the operating room on ice in cold Dulbecco’s modified Eagle medium (Hyclone Technologies, South Logan, UT, USA). Then, the tissue samples were washed in PBS (Hyclone Technologies, South Logan, UT, USA) and a surgical blade was used to mince the tissue into 2-mm-sized pieces. Next, 0.1% type 1 collagenase (Sigma, C-0130-1G, St Louis, MO, USA) was added twice volume to the tissue, and the samples were incubated in a shaking incubator at 37 °C for 1 to 2 h. The digested tumor was then filtered through a 100 μm mesh filter and collected in a 50 ml tube. After centrifugation at 1500 rpm for 10 min, the tumor cells were again washed with PBS, and finally seeded onto 100-mm culture dishes. For culture, we exchanged the media every 3–4 days. MSCs derived human adipose tissue were cultured and maintained in DMEM/12, while DDLPS cell lines and DDLPS PDCs were maintained in DMEM/High (Invitrogen, Carlsbad, CA, USA) supplemented with 10% fetal bovine serum (FBS; HyClone) and antibiotic-antimycotic solution (1:100; HyClone). DDLPS PDCs were characterized using fluorescence in situ hybridization (FISH), karyotyping, and array comparative genomic hybridization (CGH) [29]. Well-characterized cells that could represent patient tumors were used in this study.

### Cell survival and apoptosis analysis

To analyze rhTRAIL-induced cell death, human sarcoma cell lines (SW872, HT1080 LPS246, LPS224) and DDLPS PDCs (11GS-013, 11GS-079, 11GS-099, 11GS-106, 14GS-026, 11GS-076) were treated with increasing concentrations of recombinant TRAIL protein (0–100 ng/ml) combined with c-Met inhibitors (PHA-665752: PHA; PF02341066: PF) (0–10 μM),. Cell viability was measured after 48 h of treatment using a Cell Counting Kit-8 (CCK8; Dojindo Molecular Technologies, Rockville, MD, USA). The drug effects were determined using a high sensitivity cell proliferation/cytotoxicity assay based on the determination of WST-8. All procedures were performed according to the manufacturer’s instructions. Briefly, cells were seeded in 96-well microtiter plates (5 × 10^3^ cells/well) and grown in DMEM were incubated with either PBS (as a control group) or the indicated drug for 48 h. Plates were incubated under standard cell culture conditions. Tests were performed in triplicate; four wells per plate with medium only served as blanks, and three wells with untreated cells served as controls. After being loaded into SpectraMax® M 4 Multi-Mode Microplate Readers (Molecular Devices, LLC, Sunnyvale, CA, USA), the plates were measured at 450 nm. Statistical analysis of the results was performed using an unpaired Student’s t-test. To determine whether the c-Met inhibitors affected TRAIL sensitivity, DDLPS cells were subjected to the following treatment (Fig. 4):

1. combination treatment with rhTRAIL and PF for 48 h;
2. treatment with rhTRAIL or with PF alone for 48 h;
3. treatment with PF for 24 h, followed by rhTRAIL for 24 h;
4. treatment with rhTRAIL for 24 h, followed by PF for 24 h;

Next, induced apoptosis was assessed via flow cytometry using annexin V and 7-aminoactinomycin D (7-AAD; BD Biosciences, BD 1 Becton Drive Franklin Lakes, NJ, USA).

#### IC50 and Combination Index (CI)

The IC50 values of each agent were determined using a sigmoidal dose-response (variable slope) curve using Graph Pad Prism software. The concentration of each drug was set for use in combination experiments (Additional file 1: Table S1). The data was then analyzed using the CompuSyn Combination Index (CI) methodology, which was used to evaluate the interactions of several drug effects [30]. The CI Value quantitatively defines synergism (CI < 1), additive effect (CI = 1) and antagonism (CI > 1). This method for assessing synergy takes into account both the potency (Dm or inhibitory concentration 50% (IC50)) and shape of the dose-effect curve [31]. The ratio of rhTRAIL to the PHA or PF was referred to the concentration values where covered the dose-effective curve (Additional file 2: Table S2).

### Flow cytometry

Cells were stained with PE-conjugated antibodies against human decoy receptors 1 (DcR1) and 2 (DcR2), death receptors 4 (DR4) and 5 (DR5), and c-Met (R&D Systems, Minneapolis, MN, USA). Cells were incubated with 2 μg/mL of primary anti-hTRAILR1 (DR4), anti-hTRAILR2 (DR5), anti-hTRAILR3 (DcR1), anti-hTRAILR4 (DcR2) mouse IgG1-PE conjugated antibodies for 1 h at 4 °C for determination of the surface levels of TRAIL receptors. For analysis of surface expression of c-Met, cells were incubated with 1 μg/mL of primary anti-HGFR mouse IgG1-PE-conjugated antibody for 1 h at 4 °C. After, cells were washed with PBS and incubated with a PE-conjugated secondary antibody (BD Biosciences Franklin Lakes, NJ, USA) for 30 min at 4 °C. For the respective isotype controls, cells were stained with BD Pharmingen™ PE Mouse IgG1, κ Isotype Control. Cells were analyzed using a FACS Calibur instrument (BD Biosciences, Franklin Lakes, NJ, USA). Data were analyzed using the CellQuest program (BD Biosciences). All procedures were repeated at least three times.

#### Detection of DR5 mRNA expression by real-time quantitative PCR

Total RNA preparation and the RT reaction were carried out as described previously [32]. PCRs were performed using an ABI Prism 7900HT according to the manufacturer’s instructions. Amplification of specific PCR products was detected using the SYBR Green PCR Master Mix (Applied Biosystems, Waltham, Massachusetts, USA). The primers designed in this study were as follows: DR5 forward primer, 5′- GAGCTAAGTCCCTGCACCAC -3′; DR5 reverse primer, 5′- AATCACCGACCTTGACCATC -3′; GAPDH forward primer, 5′-CGGCGACGACCC ATTCGAAC-3′; and GAPDH reverse primer, 5′-GAATCGAACCCTGATTCCCCG TC-3′. RNA samples were normalized to the level of GAPDH mRNA. The real-time PCR was performed in triplicate in a total reaction volume of 25 μL containing 12.5 μL of SYBR Green PCR Master Mix, 300 nM forward and reverse primers, 11 μL of distilled H2O, and 1 μL of cDNA from each sample. Samples were heated for 10 min at 95 °C and amplified for 40 cycles of 15 s at 95 °C and 60 s at 60 °C. Quantification was performed using the 2-ΔΔCt method. The Ct value is defined as the cycle of the PCR where the amplified product is detected. The ΔCt was obtained by subtracting the Ct value of housekeeping gene (GAPDH) from that of interest gene (DR5).

#### Western blotting

Western blot analyses were performed as previously described [33]. Briefly, cells were washed twice with PBS and then lysed via sonication in lysis buffer (Intron, Seoul, Korea). The samples were separated on 10–15% SDS-PAGE gels, and then transferred to nitrocellulose membranes (Protran BA83; Whatman). Immunoblotting was performed using the following primary antibodies and dilutions: anti-Capase 8 (1:1000; Cell Signaling, Danvers, MA, USA), anti-Caspase 3 (1:1000; Cell Signaling, Danvers, MA, USA), anti-Caspase 7 (1:1000; Cell Signaling, Danvers, MA, USA), anti-cleaved Caspase 7 (1:1000; Cell Signaling, Danvers, MA, USA), PARP (1:1000; Cell Signaling, Danvers, MA, USA), BCL2 (1:1000; Cell Signaling, Danvers, MA, USA), DR4 (1:1000; Abcam, Cambridge, MA), DR5 (1:000; Abcam, Cambridge, MA, USA) and anti-beta actin (1:10000; Sigma, St. Louis, MO, USA). Horseradish peroxidase-labeled rabbit anti-mouse (Abcam, diluted 1:5000, Cambridge, MA, USA) and goat anti-rabbit (Santa Cruz, diluted 1:2000, Finnell Street, Dallas, TX, USA) secondary antibodies were used. The proteins were visualized using an ECL detection system (Ab Frontier, Seoul, Korea).

#### Small interfering RNAs

The siRNA sequences against human DR5 and nonsilencing were chemically synthesized by Bioneer (Daejoun, Korea republic). The siRNA sequences were designed from AccuTarget Genome-wide Predesigned siRNA library (Bioneer, Daejoun, Korea republic). LPS224 cell was plated on 6-well plates at 3 × 10^5^ cells per well and transfected with 100 pmol of siRNA duplex per well using Lipofectamine 2000 (Invitrogen, Waltham, Massachusetts, USA) as the manufacturer’s recommendations. Cells were harvested 48 h after transfection with PF and/ or rhTRAIL treatment.

### Statistical analysis

One-way analysis of variance (ANOVA) was used to compare cell viability of LPS cells in each condition, followed by Tukey post hoc multiple comparisons test. All data were presented as the mean ± standard deviation (SD). Statistical analysis was performed using Graph Pad Prism (La Jolla, CA, USA), and a probability value of < 0.05 was considered statistically significant. *, *P* < 0.05; **, *P* < 0.01; ***, *P* < 0.001; ****, *P* < 0.0001.

## Results

### Sarcoma cell lines exhibited varying sensitivities to rhTRAIL

**Fig. 1:**
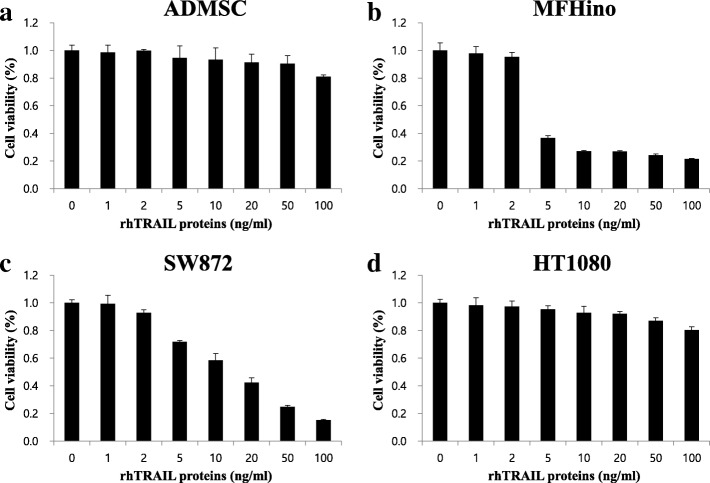
Efficacy of treatment with rhTRAIL in sarcoma cell lines. Cell viability of ADMSCs (**a**), MFH-ino (**b**), SW872 (**c**), and HT1080 (**d**) after 48 h of incubation with serial dilutions of rhTRAIL protein (0–10 ng/ml)

To evaluate the TRAIL sensitivities of various sarcoma cell lines, undifferentiated pleomorphic sarcoma (UPS; MFHino), liposarcoma (LPS; SW872), and fibrosarcoma (FS; HT1080) cell lines were treated with increasing doses of rhTRAIL. MFHino and SW872 cells were found to be highly sensitive to low doses of rhTRAIL, whereas HT1080 cells showed TRAIL resistance approaching that of normal control cells and ADMSCs, even under high rhTRAIL doses (Fig. 1: Additional file 1: Table S1).

### C-met inhibitors combined with rhTRAIL enhanced cell death in sarcoma cell lines

To determine whether c-Met inhibitors enhanced sensitivity to TRAIL in sarcoma cell lines, MFH-ino cells were treated with increasing doses of one of two c-Met inhibitors, PHA or PF, in combination with rhTRAIL. Our results demonstrated that treatment with rhTRAIL alone or in combination with either of the two c-Met inhibitors resulted in similar cell viability in MFH-ino cells, with up to 80% cell death after treatment for 48 h (Fig. 1b, Additional file 3: Figure S1b). Combined treatment with c-Met inhibitors and rhTRAIL resulted in diminishing viability in SW872 cells (80–90%) compared to treatment with rhTRAIL alone (Fig. 1c, Additional file 3: Figure S1c). When we co-treated HT1080 cells, which demonstrated high resistance to various concentrations of rhTRAIL (1–10 ng/ml), with PHA or PF, we observed that the combination treatment could overcome this TRAIL resistance. Meanwhile, treatment with doses higher than 5 μM of PHA or 5 μM of PF dramatically decreased cell viability. (Fig. 1d, Additional file 2: Table S2: Additional file 3: Figure S1d). Previously, we showed that PHA had strong in vivo toxicity (data not shown); hence, we selected PF as a candidate drug for combination therapy with rhTRAIL treatment. Using this dual treatment approach, we then tried to identify the relationship between cell viability and the upregulation of death receptors induced by PF.

### The c-met inhibitor PF induced upregulation of DR5 in sarcoma cells

We analyzed the expression of death receptors and c-Met via flow cytometry. The analysis revealed that the expression levels of death receptor 5 (DR5) were 82.8% in MFH-ino cells (Additional file 4: Figure S2a), 16.6% in SW872 cells (Additional file 4: Figure S2b), and 0.4% in HT1080 cells (Additional file 4: Figure S2c). c-Met receptor showed very high expression levels in the three sarcoma cell lines, at values of 99.9% in MFH-ino cells, 99.8% in SW872 cells, and 99.7% in HT1080 cells. From these results, we found there was a correlation between the expression levels of DR5 and cellular sensitivity to TRAIL. On the other hand, c-Met expression was not shown to be correlated with TRAIL sensitivity. Even the TRAIL-resistant HT1080 cells displayed high expression levels of c-Met.

To further verify the enhanced anti-cancer efficacy of rhTRAIL and PF combination treatment, SW872 cells were treated with 5 ng/ml rhTRAIL combined with 5 μM of PF, after which apoptosis was assessed via annexin V and 7-AAD assays. After 48 h of incubation, SW872 cells showed markedly higher rates of apoptotic cell death, whereas ADMSCs were found to be resistant to the combination treatment (Additional file 5: Figure S3).

**Fig. 2:**
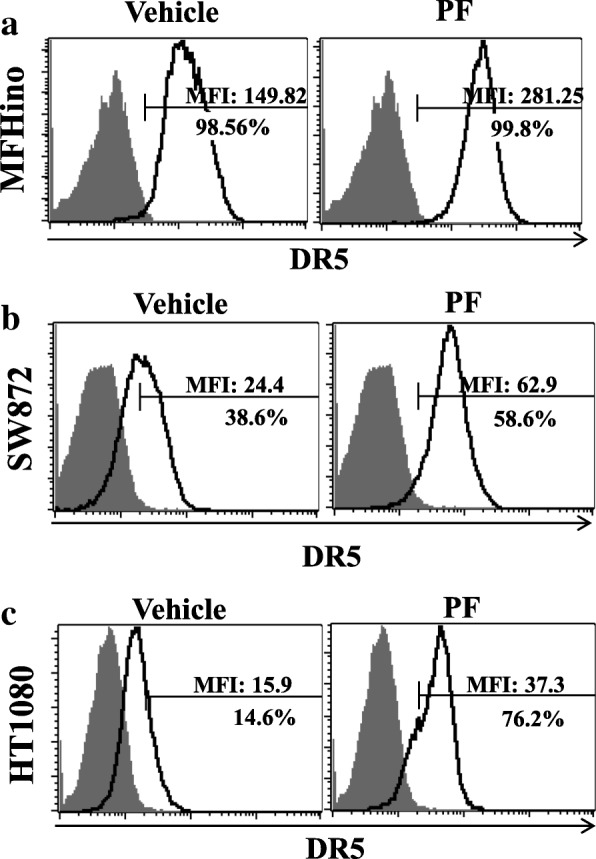
Treatment with the c-Met inhibitor PF induced DR5 upregulation. DR5 expression levels in MFH-ino (**a**), SW872 (**b**), and HT1080 (**c**) cells after 48 h of incubation with PF as measured via flow cytometry. The left panel shows control cells with DMSO (shaded gray histogram) and the right panel shows cells treated with 10 μM PF (bold black open histogram)

Next, we further aimed to elucidate the mechanisms underlying the effects of combination treatment with the c-Met inhibitor PF and rhTRAIL in sarcoma cell lines (MFH-ino, SW872, and HT1080). To this end, we analyzed DR5 expression levels using flow cytometry after incubation with PF for 24 h. The analysis showed that DR5 was upregulated when these cell lines were pretreated with PF compared to non-pretreated groups. From these data, we concluded that the increased apoptosis seen after combination PF/ rhTRAIL treatment, when compared with treatment with rhTRAIL alone, was mainly due to the activation of the death receptor DR5 induced by PF (Fig. 2).

### Combined therapy with the c-met inhibitor PF and rhTRAIL reduced cell survival of liposarcoma patient-derived cells

**Fig. 3:**
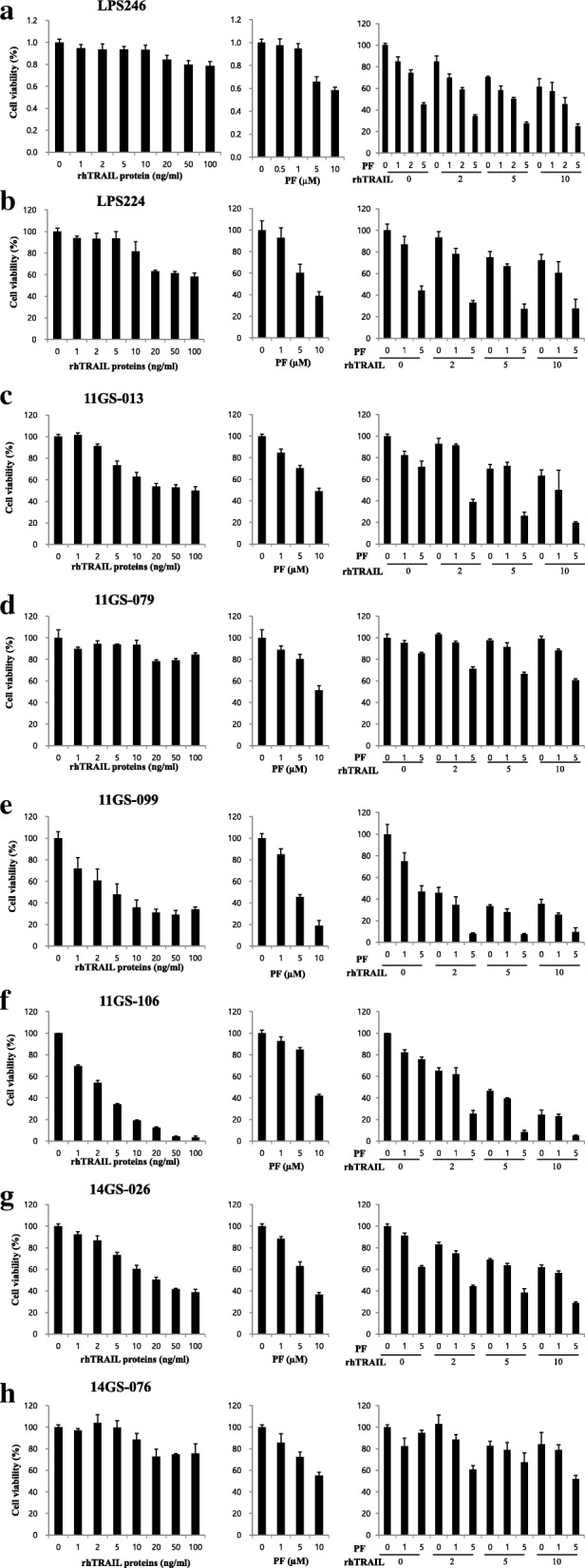
Efficacy of tumor cell suppression through combined treatment with the c-Met inhibitor PF and rhTRAIL in DDLPS PDCs. Combination treatment with PF and rhTRAIL suppressed cell viability effectively in the DDLPS established cell lines: LPS246 (**a**) and LPS224 (**b**); and in the DDLPS PDCs: 11GS-013 (**c**), 11GS-079 (**d**), 11GS-099 (**e**), 11GS-106 (**f**), 14GS-026 (**g**), and 14GS-076 (**h**)

For validating the tumor suppressive efficacy of PF combined with rhTRAIL, we next attempted to focus our analysis on DDLPS PDCs. To this end, we established PDCs from patient tumor tissues and characterized them via FISH using the MDM2 probe and karyotyping. After well-characterized PDCs were selected, we used them to validate the efficacy of the combined treatment. Our results showed that each set of PDCs had a different response to TRAIL. In particular, 11GS-099 (Fig. 3e), 11GS-106 (Fig. 3f), and 14GS-026 (Fig. 3g) cells were sensitive to rhTRAIL treatment, whereas LPS246 (Fig. 3a), LPS224 (Fig. 3b), 11GS-013 (Fig. 3c), 11GS079 (Fig. 3d), and 14GS-076 (Fig. 3h) cells showed resistance to rhTRAIL. However, combination treatment with a c-Met inhibitor (PF) and rhTRAIL increased cell death by about 40% in all TRAIL-resistant cells (Additional file 6: Figure S4). These results revealed that combined treatment with rhTRAIL and PF can potentially overcome TRAIL resistance in DDLPS PDCs.

### The c-met inhibitor PF promoted sensitization of TRAIL-resistant liposarcoma patient-derived cells

**Fig. 4:**
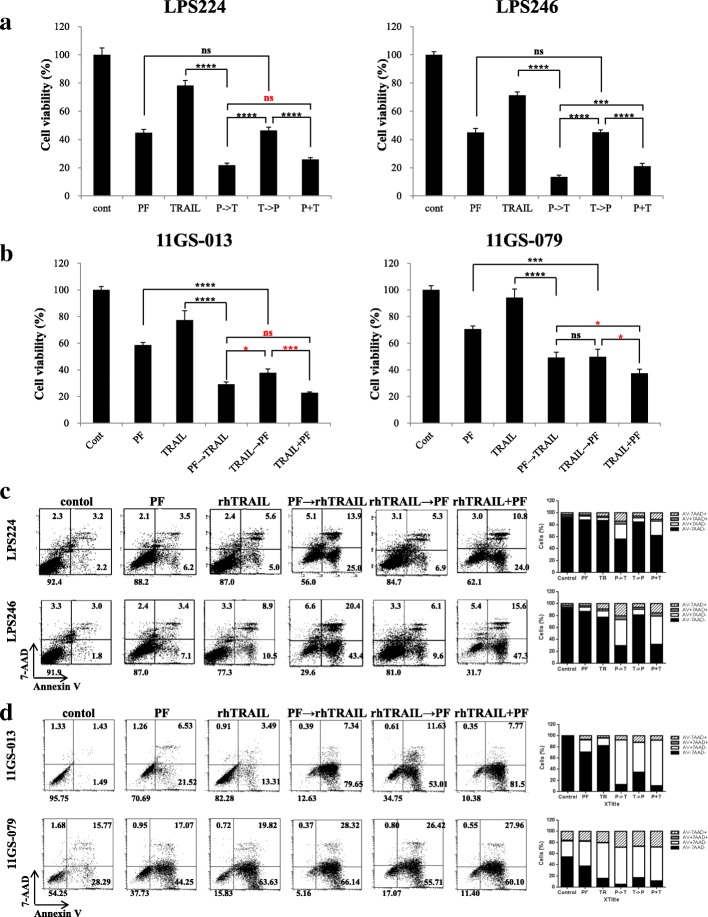
The c-Met inhibitor PF enhanced TRAIL-mediated apoptosis in liposarcoma. PF enhances TRAIL-mediated apoptosis in DDLPS cell lines and PDCs. Shown are the cell viabilities of the established cell lines (**a**) LPS224 and LPS246 and the PDCs (**b**) 11GS-013 and 11GS-079 after 48 h of incubation with 5 μM PF and 5 ng/ml rhTRAIL under the following treatment schemes: negative control, PF alone for 48 h; rhTRAIL alone for 48 h; PF for 24 h followed by rhTRAIL for 24 h; rhTRAIL for 24 h followed by PF for 24 h; and concurrent treatment with PF and rhTRAIL for 48 h. We analyzed apoptosis using annexin V and 7-AAD (**c** and **d**)

We examined the anti-cancer efficacy of sequential treatment with PF followed by rhTRAIL, or rhTRAIL followed by PF, in LPS cell lines (LPS246 and LPS 224; Fig. 4a) and PDCs (11GS-013 and 11GS-079 Fig. 4b), and analyzed apoptosis rates using annexin V and 7-AAD assays (Fig. 4c and d). Notably, pre-treatment with PF for 24 h, followed by rhTRAIL for 24 h, reduced not only cell viability, but also induced apoptosis at a significantly higher rate compared to that of other treatment groups across all DDLPS cell lines and PDCs (Fig. 4). However, the Caspase-8, Caspase-3 in pre-treated PF group was not different from those in groups with combined treatment (Additional file 7: Figure S5).

### The c-met inhibitor PF increased DR5 expression and sensitized apoptosis in DDLPS cells

**Fig. 5:**
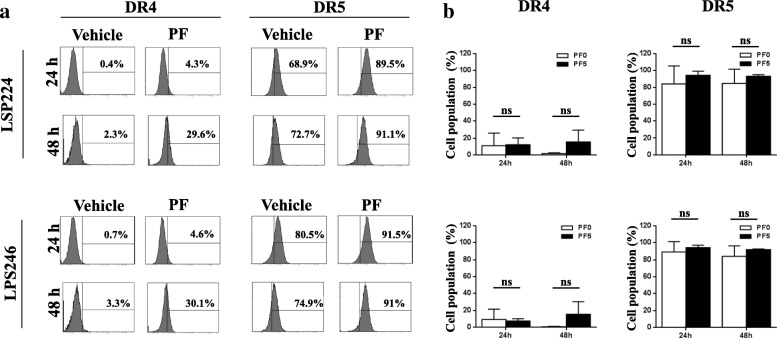
Death receptor 5 was upregulated in DDLPS cells upon PF c-Met inhibitor treatment. Expression levels of DR4 (**a**) and DR5 (**b**) in LPS224 and LPS246 cells after 24 h and 48 h of incubation with PF measured via FACS. The left panel shows control cells with DMSO (shaded gray histogram) and the right panel shows cells treated with 10 μM PF (bold black open histogram)

To understand the effect of pre-treatment of cells with PF, we analyzed the expression levels of certain death receptors in DDLPS cell lines (LPS224 and LPS246). Flow cytometry analysis showed that both DR4 and DR5 were upregulated in cell lines pre-treated with PF. In particular, DR5, which is known to be one of the critical factors for the TRAIL-induced apoptosis pathway, was upregulated by up to 89.5%, whereas DR4 was upregulated by just 4.6% after 24 h of incubation with PF. This increase in DR5 expression was detected until 48 h after PF treatment, thereby demonstrating that the effects of DR5 up-regulation was observed upon treatment with the c-Met inhibitor PF even though they have almost same cell populations (Fig. 5a and b). These results showed that inhibition of c-Met could potentially induce DR5 expression in not only transcription level but also in translational quantity, which in turn could enhance TRAIL-induced apoptosis in DDLPS cells (Additional file 8: Figure S6 and Additional file 9: Figure S7).

### DR5 is needed for the cell death by c-met inhibitor in DDLPS

For the purpose of evaluating these results, we knocked down DR5 in liposarcoma cell lines and PDCs. In the DR5 knocked-down groups, we could not detect TRAIL-induced apoptosis. Even when DR5 knocked-down cells were treated PF, there was minimal induction of apoptosis. From these results, we confirmed that DR5 is important for apoptosis induced by PF. This mechanism may explain why cells treated with PF were more susceptible to cell death (Additional file 10: Figure S8). We hypothesized that there was a relationship between DR5 and c-Met expression. Our data showed that the inhibition of c-Met led to the activation of DR5, and cell death was commensurately and significantly induced. Further study is needed to fully elucidate TRAIL-related anti- or pro-cell death factors, especially those related to c-Met, which is over activated in liposarcoma [34].

Here, we demonstrated conclusively that there was suppression of c-Met after c-Met inhibitor treatment, and that TRAIL-induced apoptosis involved c-Met in liposarcoma cell lines.

## Discussion

In this study, we demonstrated that combined therapy with a c-Met inhibitor and TRAIL could significantly reduce survival of liposarcoma cells. Our results showed that treatment with a c-Met inhibitor sensitized cells to TRAIL-induced apoptosis via DR5 upregulation in DDLPS cell lines and PDCs, which have TRAIL-resistant characteristics.

TRAIL is considered to be a targeted anti-cancer agent because it induces apoptosis in several kinds of cancers while leaving normal cells intact [35]. TRAIL-based drugs have been developed that target death receptors, specifically DR4 and DR5, in STS [6]. Meanwhile, multiple studies have reported that many types of cancer cells exhibit various TRAIL sensitivities [36]. Here, we assessed the TRAIL sensitivities of several sarcoma cell lines and DDLPS PDCs. Our data showed that treatment with TRAIL induced significant caspase-8-mediated apoptosis in these cell lines, except HT1080. Validation studies using established DDLPS cell lines and DDLPS patient-derived cells (PDCs) showed that each cell line had varying susceptibilities to TRAIL treatment. Several previous reports have indicated that targeting the c-Met pathway could overcome resistance to other drugs, such as TRAIL, and synergistically induce apoptosis in various kinds of tumor [37–43]. Consistent with those reports, our results showed that a c-Met inhibitor enhanced DR5 expression in STS cells [7]. In our study, treatment with the c-Met inhibitor PF followed by TRAIL resulted in significant induction of apoptosis in liposarcoma cells. However, treatment with TRAIL followed by PF did not result in any changes in cell viability when compared to treatment with PF alone; therefore, we hypothesize that PF upregulates DR5 expression and promotes TRAIL-induced apoptosis.

It was demonstrated that c-Met contributes to TRAIL sensitivity in brain tumor cells and non-small cell lung cancer cell lines, which has implications for developing effective therapies for patients. They found a direct correlation between the c-Met expression level and TRAIL resistance, and also showed that the knockdown of the c-Met protein or targeting the c-MET using small interference RNA, sensitized tumor cells to TRAIL-mediated apoptosis by interrupting the interaction between c-Met and TRAIL cognate death receptors [24, 44]. Similar to Wanlu’s data, our data revealed that increased DR5 expression was observed 48 h after c-Met inhibitor treatment in PDCs (Fig. 5a and b: Additional file 8: Figure S6 and Additional file 9: Figure S7) [24]. Even in 11GS079 PDCs, which had low expression levels of DR5 and high expression of c-Met (Additional file 11: Figure S9), suppression of cell growth was induced significantly by treatment with PF followed by TRAIL or simultaneous combination treatment compared to that seen after treatment with PF or TRAIL alone (Fig. 4). This may be due to the following reasons: 1) the DR5 pathway is strongly related to the activity of c-Met; or 2) inhibition of a tyrosine kinase associated with c-Met becomes enhancing DR5 expression and inducing the pro-apoptotic effect. To investigate this connection, we knocked down DR5 and then treated cells with PF and TRAIL. As a result, DR5 expression was remarkably increased following treatment with PF in DR5 wild type cells. On the contrary, in DR5-KD cells, the expression level of DR5 was very small; also it was not detected cleaved form of caspase-3, caspase-8 and caspasse-7 after PF treatment. These results suggested that the increase in apoptosis seen following pre-treatment with PF was mainly due to induction of DR5 (Additional file 10: Figure S8).

In this study, we validated the efficacy of combined c-Met inhibitor and TRAIL treatment in DDLPS PDCs that we established and characterized. This approach is highly effective for personalized medicine. Moreover, validation of therapeutic drugs using patient tumor-derived cells is an approach that can overcome the differences in drug responses seen between preclinical and clinical studies, thereby increasing the possibility of successful clinical application. The validation of a treatment’s anticancer effect using PDCs has broadly been found to be very effective. However, the use of patient-derived xenograft mouse (PDX) models give even more confidence to a treatment’s anti-cancer effect, and allows for better insight into potential drug sensitivities for each patient [45]. PDXs are created by grafting fresh tumor tissues onto mice, and provide a suitable tumor microenvironment, as well as tumor tissue heterogeneity [46]. We are currently establishing PDX models to explore the drug efficacies of combination therapy. These are particularly useful tools for elucidating the mechanisms behind the enhancement of DRs caused by c-MET inhibitors and TRAIL-induced apoptosis. Hence, it may serve as a primary tool for verifying the availability of medications for patients in preclinical stages.

## Conclusion

To our knowledge, this study is the first study to report that individual DDLPS PDCs exhibit different responses to TRAIL. In addition, targeted treatment using combination therapy with c-Met inhibitors and TRAIL effectively sensitized cells to TRAIL and displayed anti-cancer effects in aggressive DDLPS with high drug resistance, thus leading to high rates of cell death. We demonstrated that this combined treatment approach is promising for clinical trials using DDLPS patients who currently have access to only a few available standard treatments.

## Additional files

Additional file 1: **Table S1.** IC50 values in STS cell lines and DDLPS PDCs. (DOCX 19 kb)

Additional file 2: **Table S2.** Combination index (CI) values. (DOCX 26 kb)

Additional file 3: **Figure S1.** Combined efficacy of c-Met inhibitor and TRAIL in normal ADMSC and STS cells. We examined the sensitivity of STS cell lines to rhTRAIL proteins. Cell viability of ADMSCs (a), MFHino (b), SW872 (c), and HT1080 (d) following 48 h of incubation with serial dilutions of rhTRAIL protein (0–100 ng/ml) and c-Met inhibitors PHA665752 (left panel), or PF02341066 (right panel) (0–20 μM). (PPTX 126 kb)

Additional file 4: **Figure S2.** Expression levels of death receptors and c-Met receptor expression in STS cell lines. TRAIL receptors, decoy receptor 1 (DcR1), decoy receptor 2, (DcR2), DR4, DR5, and c-Met expression levels in MFHino (a) SW872 (b), and HT1080 (c) cells, as analyzed by flow cytometry (isotype: shaded gray histogram; each receptors: bold black open histogram). (PPTX 129 kb)

Additional file 5: **Figure S3.** c-Met inhibitor, PF and TRAIL treatment induced apoptosis in DDLPS cell lines. Apoptosis were induced through treatment with the c-Met inhibitor PF and rhTRAIL in liposarcoma cell lines. FACS plot showing apoptosis in ADMSCs (A) and SW872 cells (B) following 48 h of incubation with rhTRAIL (0, 2, 5 ng/ml) and PF (0, 5 μM) using annexin V and 7AAD. (PPTX 343 kb)

Additional file 6: **Figure S4.** Efficacy of tumor cell suppression through combined treatment with the c-Met inhibitor, PF and rhTRAIL in DDLPS PDCs. Human liposarcoma cells were treated with PF (5 μM) and rhTRAIL (5 ng/ml) for 48 h. Cell viability was analyzed by CCK8: (a) rhTRAIL only (5 ng/ml) (b) PF only (5 μM) (c) combination treatment with PF (5 μM) and rhTRAIL (5 ng/ml). (PPTX 102 kb)

Additional file 7: **Figure S5.** Cell death was induced by PF and/ or rhTRAIL treatment. Representative Western blots of caspase 3, caspase 7, caspase 8, Bcl2, PARP, DR4 and DR5 were shown. Membranes were re-probed for ACTB expression to show that similar amounts of protein were loaded in each lane for LPS246 cells (a) and 11GS079 PDC (b). (1) primary treatment, (2) secondary treatment. (PPTX 307 kb)

Additional file 8: **Figure S6.** Induced expression levels of DR5 mRNA in DDLPS cells by PF and/ or rhTRAIL treatment. DR5 mRNA expression levels were detected in DDLPS cells after treatment with PF and/ or rhTRAIL. LPS246 and 11GS079 cells were treated with DMSO (as control), PF (5 μM), rhTRAIL (5 ng/mL) and PF (5 μM) with rhTRAIL (5 ng/mL) simultaneously for 48 h. RNA samples were isolated and subjected to real-time PCR analysis. Data were normalized GAPDH level and presented as fold changes in fluorescence density compared to that of the control group. Data are shown as the mean ± SD. *, *P* < 0.05; ***, *P* < 0.001 versus control. (PPTX 68 kb)

Additional file 9: **Figure S7.** Death receptor was up-regulated by c-Met inhibitor, PF. Representative Western blot results of Bcl2, DR4 and DR5 were shown. Membranes were re-probed for ACTB expression to show that similar amounts of protein were loaded in each lane in LPS246 cells (a) and 11GS079 PDCs (b). (1) primary treatment, (2) secondary treatment. (PPTX 156 kb)

Additional file 10: **Figure S8.** Effect of apoptosis by combination treatment with PF and/ or rhTRAIL and combined with DR5 siRNA. To determine the direct roles of DR5 in PF-induced TRAIL sensitization, LPS224 cells were treated with DR5 siRNA, followed by co-treatment with PF (5 μM) and rhTRAIL (5 ng/ml) for 48 h. Representative Western blots of caspase-3, caspase-7 (a), and caspase-8 (b) were shown. (PPTX 275 kb)

Additional file 11: **Figure S9.** c-Met and rhTRAIL receptor expression levels in DDLPS. PDCs. c-Met inhibitor PF upregulated expression levels of c-Met in DDLPS PDCs. The expression levels of DcR1, DcR2, DR4, DR5, and c-Met were analyzed by flow cytometry after DMSO (vehicle: shaded gray histogram) and PF (5 μM: bold black open histogram) treatment for 48 h, as shown in the upper column (a). c-Met expression levels in LPS224, LPS246, 11GS-013, 11GS-079, 11GS-099, 11GS-106 and 11GS-076 cells were analyzed by flow cytometry (b). (PPTX 513 kb)

## Abbreviations

ADMSC: Adipose tissue derived mesenchymal stem cell
DDLPS: Dedifferentiated liposarcoma
DR5: Death receptor 5
LPS: Liposarcoma
PDCs: DDLPS patient-derived cells
PF: PF02341066 (crizotinib)
PHA: PHA665257
rhTRAIL: human recombinant TNF related apoptosis inducing ligand
STS: Soft tissue sarcoma
TRAIL: Tumor necrosis factor-related apoptosis-inducing ligand
WDLPS: Well-differentiated liposarcoma
