# Supplementary material for: Combination therapy with c-met inhibitor and TRAIL enhances apoptosis in dedifferentiated liposarcoma patient-derived cells
Source: BMC Cancer. 2019 May 24;19:496. doi: 10.1186/s12885-019-5713-2 (PMC6534902; doi:10.1186/s12885-019-5713-2)
Supplement: Supplementary file 1 — Table S1. IC50 values in STS cell lines and DDLPS PDCs. (DOCX 19 kb) [file 12885_2019_5713_MOESM1_ESM.docx]

Additional file 1: **Table S1.** IC50 values in STS cell lines and DDLPS PDCs.

|  | IC50 (nM±std) | | |
| --- | --- | --- | --- |
|  | rhTRAIL | PF | PHA |
| ADMSC | 20±0.6572 | 9.825±0.08781 | 10.09±0.1012 |
| MFHino | 4.285±0.1186 | 9.908±0.1841 | 10.81±0.2961 |
| SW872 | 9.977±0.3928 | 9.949±0.2339 | 10.17±0.04270 |
| HT1080 | 20.26±0.5050 | 5.138±0.3470 | 9.827±0.1572 |
| LPS224 | 5.393±1.495 | 5.842±0.5897 |  |
| LPS246 | 10.74±0.7641 | 4.371±0.2402 |  |
| 11GS 013 | 4.884±0.2466 | 5.122±0.1399 |  |
| 11GS 079 | 11.11±2.030 | 5.316±0.4252 |  |
| 11GS 099 | 0.7337±0.3408 | 4.762±0.5843 |  |
| 11GS 106 | 1.915±0.3615 | 5.572±0.2879 |  |
| 14GS 076 | 2.276±0.7440 | 5.123±0.7124 |  |

Comparison of IC50 values in MSC, STS cell lines and DDLPS PDCs for rhTRAIL, PHA and PF for CCK8 assays. IC50 values were the concentration of compound required to inhibit the cell growth by 50% compared with an untreated control. Values are averages of three independent experiments; std: standard deviation.
