# Supplementary material for: Combination therapy with c-met inhibitor and TRAIL enhances apoptosis in dedifferentiated liposarcoma patient-derived cells
Source: BMC Cancer. 2019 May 24;19:496. doi: 10.1186/s12885-019-5713-2 (PMC6534902; doi:10.1186/s12885-019-5713-2)
Supplement: Supplementary file 2 — Table S2. Combination index (CI) values. (DOCX 26 kb) [file 12885_2019_5713_MOESM2_ESM.docx]

Additional file 2: **Table S2.** Combination index (CI) values.

| ADMSC |  | PHA, µM with rhTRAIL, ng/mL | | | |
| --- | --- | --- | --- | --- | --- |
| PHA / rhTRAIL**#** | CI | ED50 | ED75 | ED90 | ED95 |
| **1:1** |  | **9.59E-4** | **0.00104** | **0.00113** | **0.0012** |
| 1:2 |  | 8.78E-5 | 3.35E-4 | 0.00128 | 0.00319 |
| 1:10 |  | 1.65E-5 | 6.50E-5 | 2.56E-4 | 6.54E-4 |
| 2:1 |  | 2.67E-4 | 7.02E-4 | 0.00184 | 0.00356 |

| ADMSC |  | PF, µM with rhTRAIL, ng/mL | | | |
| --- | --- | --- | --- | --- | --- |
| PF/ rhTRAIL# | CI | ED50 | ED75 | ED90 | ED95 |
| **1:1** |  | **1.78E-8** | **9.34E-8** | **4.90E-7** | **1.51E-6** |
| 1:2 |  | 2.77E-9 | 5.14E-8 | 9.52E-7 | 6.94E-06 |
| 1:10 |  | 2.61E-9 | 4.99E-8 | 9.51E-7 | 7.07E-06 |
| 2:1 |  | 2.11E-9 | 2.80E-8 | 3.70E-7 | 2.15E-06 |

| MFHino |  | PHA, µM with rhTRAIL, ng/mL | | | |
| --- | --- | --- | --- | --- | --- |
| PHA / rhTRAIL# | CI | ED50 | ED75 | ED90 | ED95 |
| **1:1** |  | **9.22E-7** | **1.39E-6** | **2.11E-6** | **2.80E-6** |
| 1:2 |  | 3.63E-7 | 4.47E-7 | 5.51E-7 | 6.36E-7 |
| 1:10 |  | 2.03E-8 | 9.49E-9 | 4.45E-9 | 2.68E-9 |
| 2:1 |  | 5.88E-7 | 2.36E-7 | 9.44E-8 | 5.07E-8 |

| MFHino |  | PF, µM with rhTRAIL, ng/mL | | | |
| --- | --- | --- | --- | --- | --- |
| PF/ rhTRAIL# | CI | ED50 | ED75 | ED90 | ED95 |
| **1:1** |  | **0.01403** | **0.01502** | **0.01609** | **0.01686** |
| 1:2 |  | 0.00857 | 0.00808 | 0.00763 | 0.00733 |
| 1:10 |  | 7.35E-4 | 4.26E-4 | 2.47E-4 | 1.71E-04 |
| 2:1 |  | 0.00484 | 0.00220 | 0.00100 | 5.87E-04 |

| SW872 |  | PHA, µM with rhTRAIL, ng/mL | | | |
| --- | --- | --- | --- | --- | --- |
| PHA / rhTRAIL# | CI | ED50 | ED75 | ED90 | ED95 |
| **1:1** |  | **1.46E-5** | **1.87E-5** | **2.39E-5** | **2.83E-05** |
| 1:2 |  | 1.67E-5 | 2.69E-5 | 4.34E-5 | 6.01E-05 |
| 1:10 |  | 9.54E-8 | 1.35E-8 | 1.92E-9 | 5.1E-10 |
| 2:1 |  | 9.91E-6 | 6.55E-6 | 4.33E-6 | 3.27E-6 |

| SW872 |  | PF, µM with rhTRAIL, ng/mL | | | |
| --- | --- | --- | --- | --- | --- |
| PF/ rhTRAIL# | CI | ED50 | ED75 | ED90 | ED95 |
| **1:1** |  | **0.08721** | **0.08724** | **0.08728** | **0.0873** |
| 1:2 |  | 0.05356 | 0.04608 | 0.03966 | 0.03580 |
| 1:10 |  | 0.01720 | 0.01000 | 0.00581 | 0.00402 |
| 2:1 |  | 0.03321 | 0.01898 | 0.01085 | 0.00741 |

| HT1080 |  | PHA, µM with rhTRAIL, ng/mL | | | |
| --- | --- | --- | --- | --- | --- |
| PHA / rhTRAIL# | CI | ED50 | ED75 | ED90 | ED95 |
| **1:1** |  | **2.74E-8** | **3.98E-8** | **5.79E-8** | **7.47E-8** |
| 1:2 |  | 3.89E-8 | 8.69E-8 | 1.94E-7 | 3.35E-07 |
| 1:10 |  | 10.0E-8 | 5.73E-8 | 3.29E-8 | 2.25E-8 |
| 2:1 |  | 7.34E-8 | 2.30E-7 | 7.20E-7 | 1.57E-6 |

| HT1080 |  | PF,μM with rhTRAIL, ng/mL | | | |
| --- | --- | --- | --- | --- | --- |
| PF/ rhTRAIL# | CI | ED50 | ED75 | ED90 | ED95 |
| **1:1** |  | **0.38201** | **0.36425** | **0.34733** | **0.33627** |
| 1:2 |  | 0.38226 | 0.62457 | 1.02048 | 1.42504 |
| 1:10 |  | 1.08E-5 | 4.87E-5 | 2.20E-4 | 6.16E-04 |
| 2:1 |  | 0.62662 | 1.07785 | 1.85402 | 2.68117 |

| LPS246 |  | PF, μM with rhTRAIL, ng/mL | | | |
| --- | --- | --- | --- | --- | --- |
| PF : rhTRAIL% | CI | ED50 | ED75 | ED90 | ED95 |
| **1:1** |  | **0.12901** | **0.13083** | **0.14193** | **0.16350** |
| 1:2 |  | 0.12407 | 0.17609 | 0.28155 | 0.44187 |
| 1:5 |  | 0.09619 | 0.08524 | 0.09383 | 0.11965 |

| LPS224 |  | PF, μM with rhTRAIL, ng/mL | | | |
| --- | --- | --- | --- | --- | --- |
| PF : rhTRAIL% | CI | ED50 | ED75 | ED90 | ED95 |
| **1:1** |  | **0.15612** | **0.22207** | **0.39616** | **0.65615** |
| 1:2 |  | 0.21368 | 0.42345 | 1.02952 | 2.03716 |

| 11GS013 |  | PF, μM with rhTRAIL, ng/mL | | | |
| --- | --- | --- | --- | --- | --- |
| PF : rhTRAIL% | CI | ED50 | ED75 | ED90 | ED95 |
| **1:1** |  | **0.08042** | **0.07996** | **0.07951** | **0.07922** |
| 1:2 |  | 0.22830 | 0.72211 | 2.28448 | 5.00205 |

| 11GS079 |  | PF, μM with rhTRAIL, ng/mL | | | |
| --- | --- | --- | --- | --- | --- |
| PF : rhTRAIL% | CI | ED50 | ED75 | ED90 | ED95 |
| **1:1** |  | **0.84723** | **0.80977** | **0.82288** | **0.90116** |
| 1:2 |  | 0.51105 | 0.89953 | 1.76673 | 3.16831 |
| 11GS099 |  | PF, μM with rhTRAIL, ng/mL | | | |
| PF : rhTRAIL% | CI | ED50 | ED75 | ED90 | ED95 |
| **1:1** |  | **0.12188** | **0.19152** | **0.36267** | **0.60550** |
| 1:2 |  | 0.25124 | 0.42998 | 0.85084 | 1.42285 |

| 11GS106 |  | PF, μM with rhTRAIL, ng/mL | | | |
| --- | --- | --- | --- | --- | --- |
| PF : rhTRAIL% | CI | ED50 | ED75 | ED90 | ED95 |
| **1:1** |  | **0.46545** | **0.94278** | **1.93710** | **3.17529** |
| 1:2 |  | 2.87225 | 11.2168 | 44.1284 | 112.274 |

| 14GS076 |  | PF, μM with rhTRAIL, ng/mL | | | |
| --- | --- | --- | --- | --- | --- |
| PF : rhTRAIL% | CI | ED50 | ED75 | ED90 | ED95 |
| **1:1** |  | **0.71215** | **0.67499** | **0.66421** | **0.69218** |
| 1:2 |  | 0.78278 | 0.80344 | 0.88441 | 1.03204 |

The combination index (CI) values for combinations of rhTRAIL and PHA/ PF in MSC, STS cell lines and CI of rhTRAIL and PF in DDLPS PDCs. CI values of 1 indicate additivity, below 1 indicate synergy, and above 1 indicate antagonism. The lower the CI value the greater the degree of synergism.

#; PF:rhTRAIL, **1:1**, 5 μM: 5ng/mL, 10 μM: 10ng/mL; **1:2**, 1 μM: 2ng/mL, 5 μM: 10ng/mL; **1:10**, 0.5 μM: 5ng/mL, 1 μM: 10ng/mL; **2:1**, 10 μM: 5ng/mL, 20 μM: 10ng/mL

%; PF:rhTRAIL, **1:1**, 2 μM: 2ng/mL, 5 μM: 5ng/mL; **1:2**, 1 μM: 2ng/mL, 5μM: 10ng/mL.
