# Supplementary material for: Combination therapy with c-met inhibitor and TRAIL enhances apoptosis in dedifferentiated liposarcoma patient-derived cells
Source: BMC Cancer. 2019 May 24;19:496. doi: 10.1186/s12885-019-5713-2 (PMC6534902; doi:10.1186/s12885-019-5713-2)
Supplement: Supplementary file 3 — Figure S1. Combined efficacy of c-Met inhibitor and TRAIL in normal ADMSC and STS cells. We examined the sensitivity of STS cell lines to rhTRAIL proteins. Cell viability of ADMSCs (a), MFHino (b), SW872 (c), and HT1080 (d) following 48 h of incubation with serial dilutions of rhTRAIL protein (0–100 ng/ml) and c-Met inhibitors PHA665752 (left panel), or PF02341066 (right panel) (0–20 μM). (PPTX 126 kb) [file 12885_2019_5713_MOESM3_ESM.pptx]

## Slide 1
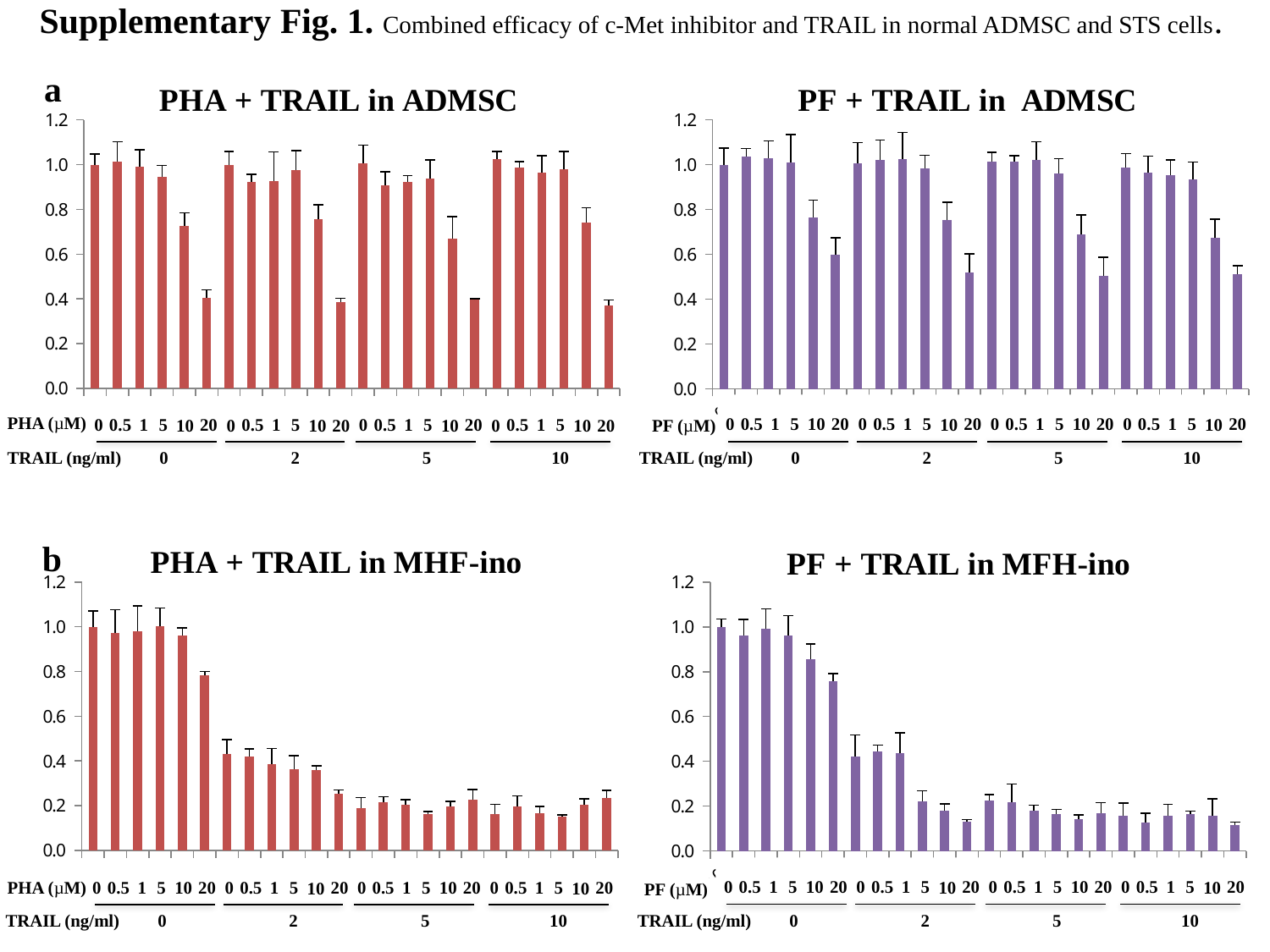

Supplementary Fig. 1. Combined efficacy of c-Met inhibitor and TRAIL in normal ADMSC and STS cells.
### Chart: PHA + TRAIL in ADMSC
| Category | PHA + TRAIL |
|---|---|
| 0+0 | 1.0 |
| 0.5+0 | 1.01534090909091 |
| 1+0 | 0.991704545454545 |
| 5+0 | 0.946136363636363 |
| 10+0 | 0.726306818181819 |
| 20+0 | 0.404431818181818 |
| 0+2 | 0.998693181818182 |
| 0.5+2 | 0.923068181818181 |
| 1+2 | 0.927329545454546 |
| 5+2 | 0.976988636363638 |
| 10+2 | 0.754375000000001 |
| 20+2 | 0.387045454545455 |
| 0+5 | 1.005965909090909 |
| 0.5+5 | 0.909147727272727 |
| 1+5 | 0.923295454545456 |
| 5+5 | 0.937102272727273 |
| 10+5 | 0.669829545454547 |
| 20+5 | 0.395284090909092 |
| 0+10 | 1.024488636363637 |
| 0.5+10 | 0.987613636363637 |
| 1+10 | 0.964261363636363 |
| 5+10 | 0.977784090909092 |
| 10+10 | 0.740340909090909 |
| 20+10 | 0.371704545454546 |
### Chart: PF + TRAIL in ADMSC
| Category | PF + TRAIL |
|---|---|
| 0+0 | 1.0 |
| 0.5+0 | 1.038128529174511 |
| 1+0 | 1.029147620328046 |
| 5+0 | 1.010432912073138 |
| 10+0 | 0.766119924710944 |
| 20+0 | 0.597042215649368 |
| 0+2 | 1.006668459263243 |
| 0.5+2 | 1.022748050551224 |
| 1+2 | 1.024576499058887 |
| 5+2 | 0.982737294971766 |
| 10+2 | 0.754934122075828 |
| 20+2 | 0.518472707717127 |
| 0+5 | 1.012637805861793 |
| 0.5+5 | 1.015756923904276 |
| 1+5 | 1.02118849152998 |
| 5+5 | 0.960849690777092 |
| 10+5 | 0.690669534821189 |
| 20+5 | 0.502608228018285 |
| 0+10 | 0.986985748857218 |
| 0.5+10 | 0.966388814197365 |
| 1+10 | 0.953105673568165 |
| 5+10 | 0.932992739983867 |
| 10+10 | 0.673137940306535 |
| 20+10 | 0.51035224522721 |a
12
12
12
PHA (µM)
PF (µM)
1
1
5
5
0.5
0.5
1
1
0
20
0
20
5
5
0.5
0.5
0
20
0
20
10
10
10
10
1
1
5
5
0.5
0.5
1
1
0
20
0
20
5
5
0.5
0.5
0
20
0
20
10
10
10
10
TRAIL (ng/ml)
2
10
TRAIL (ng/ml)
2
10
0
5
0
5
### Chart: PHA + TRAIL in MHF-ino
| Category | PHA + TRAIL |
|---|---|
| 0+0 | 1.0 |
| 0.5+0 | 0.971962301687656 |
| 1+0 | 0.980181410483368 |
| 5+0 | 1.002351929593853 |
| 10+0 | 0.960877042132417 |
| 20+0 | 0.783049247213934 |
| 0+2 | 0.429374673343113 |
| 0.5+2 | 0.41997538482289 |
| 1+2 | 0.384612142363394 |
| 5+2 | 0.363706101529176 |
| 10+2 | 0.359701920321009 |
| 20+2 | 0.254244432080657 |
| 0+5 | 0.190177532749987 |
| 0.5+5 | 0.21529850117175 |
| 1+5 | 0.204592585099388 |
| 5+5 | 0.161052383120058 |
| 10+5 | 0.198354492269823 |
| 20+5 | 0.226425910002866 |
| 0+10 | 0.161473875878812 |
| 0.5+10 | 0.197975148786944 |
| 1+10 | 0.165899549845734 |
| 5+10 | 0.149629929357814 |
| 10+10 | 0.205098376409894 |
| 20+10 | 0.23536155648846 |
### Chart: PF + TRAIL in MFH-ino
| Category | PF + TRAIL |
|---|---|
| 0+0 | 1.0 |
| 0.5+0 | 0.961037984204589 |
| 1+0 | 0.99044753666792 |
| 5+0 | 0.962241444151937 |
| 10+0 | 0.85690109063558 |
| 20+0 | 0.758518239939827 |
| 0+2 | 0.422113576532531 |
| 0.5+2 | 0.442685220007522 |
| 1+2 | 0.43490033847311 |
| 5+2 | 0.220270778488153 |
| 10+2 | 0.179616397141783 |
| 20+2 | 0.13223016171493 |
| 0+5 | 0.226739375705152 |
| 0.5+5 | 0.219330575404287 |
| 1+5 | 0.180631816472358 |
| 5+5 | 0.16615268898082 |
| 10+5 | 0.141556976306882 |
| 20+5 | 0.169612636329447 |
| 0+10 | 0.155735238811584 |
| 0.5+10 | 0.126175253854833 |
| 1+10 | 0.155321549454682 |
| 5+10 | 0.166415945844303 |
| 10+10 | 0.155885671305002 |
| 20+10 | 0.114667168108311 |b
12
12
12
PHA (µM)
PF (µM)
1
1
5
5
0.5
0.5
1
1
0
20
0
20
5
5
0.5
0.5
0
20
0
20
10
10
10
10
1
1
5
5
0.5
0.5
1
1
0
20
0
20
5
5
0.5
0.5
0
20
0
20
10
10
10
10
TRAIL (ng/ml)
2
10
TRAIL (ng/ml)
2
10
0
5
0
5

## Slide 2
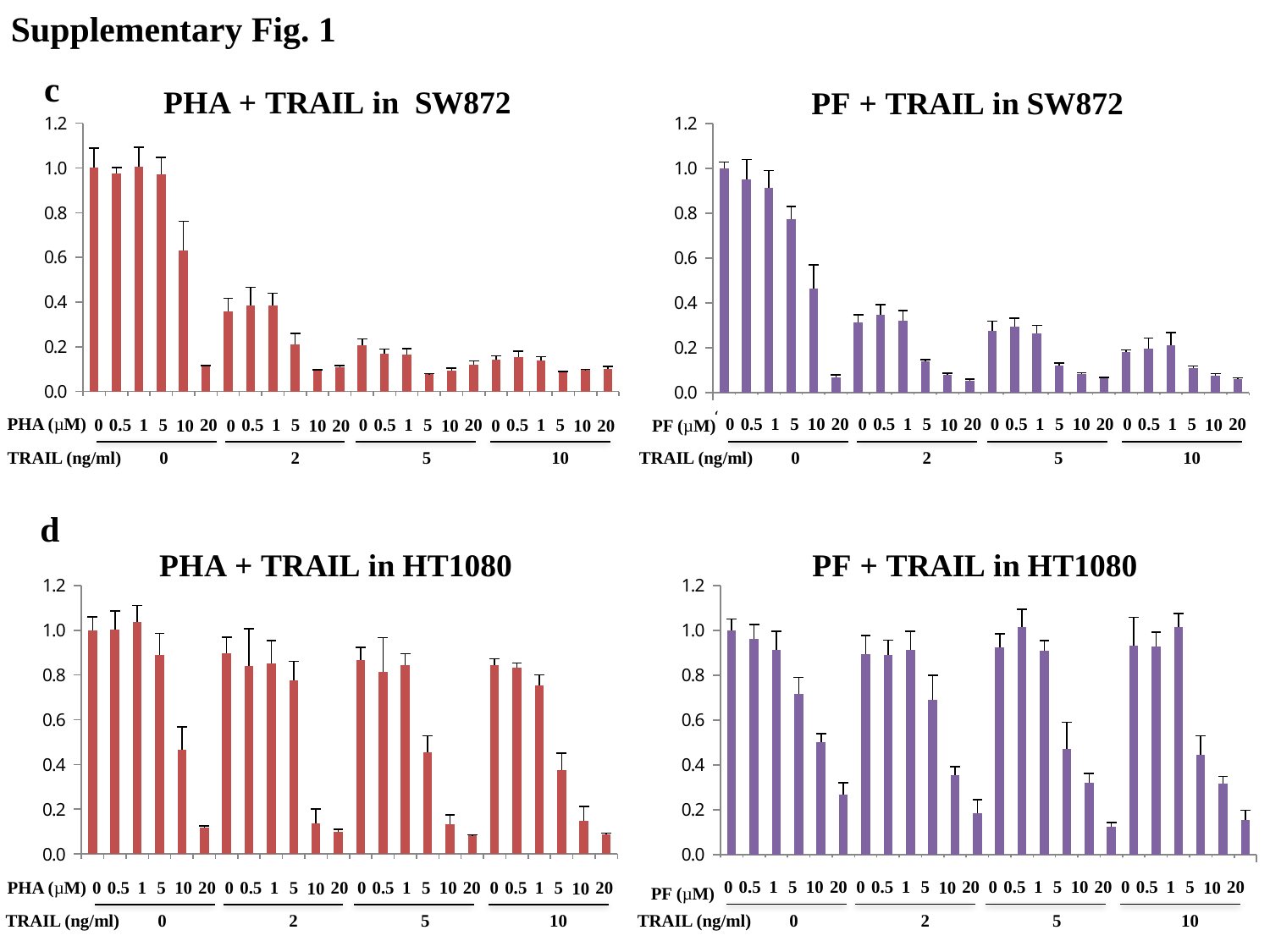

Supplementary Fig. 1
c
### Chart: PHA + TRAIL in SW872
| Category | PHA + TRAIL |
|---|---|
| 0+0 | 1.0 |
| 0.5+0 | 0.974457148156489 |
| 1+0 | 1.00699513784926 |
| 5+0 | 0.969449265378043 |
| 10+0 | 0.632172334759741 |
| 20+0 | 0.111776473549635 |
| 0+2 | 0.356990500920762 |
| 0.5+2 | 0.38542149680052 |
| 1+2 | 0.385116784356329 |
| 5+2 | 0.210953749950319 |
| 10+2 | 0.0936262105695475 |
| 20+2 | 0.109669983174574 |
| 0+5 | 0.207588664697076 |
| 0.5+5 | 0.169009419589036 |
| 1+5 | 0.164968667611717 |
| 5+5 | 0.0773969608245786 |
| 10+5 | 0.0915992103973185 |
| 20+5 | 0.11850664405612 |
| 0+10 | 0.142260966335899 |
| 0.5+10 | 0.153866535949444 |
| 1+10 | 0.139505305971039 |
| 5+10 | 0.0853857262092447 |
| 10+10 | 0.09561346564036 |
| 20+10 | 0.101429498814271 |
### Chart: PF + TRAIL in SW872
| Category | PF + TRAIL |
|---|---|
| 0+0 | 1.0 |
| 0.5+0 | 0.952037280801295 |
| 1+0 | 0.913228329777736 |
| 5+0 | 0.772789856717051 |
| 10+0 | 0.463876658647664 |
| 20+0 | 0.0673444994174875 |
| 0+2 | 0.313543037840619 |
| 0.5+2 | 0.347283373428351 |
| 1+2 | 0.319126079917693 |
| 5+2 | 0.137805819073124 |
| 10+2 | 0.0781928494696866 |
| 20+2 | 0.0541660992843419 |
| 0+5 | 0.275187992676985 |
| 0.5+5 | 0.295492714810949 |
| 1+5 | 0.263462091295599 |
| 5+5 | 0.122146066905724 |
| 10+5 | 0.0818846171304071 |
| 20+5 | 0.0618219781217375 |
| 0+10 | 0.179292814670237 |
| 0.5+10 | 0.194604572345029 |
| 1+10 | 0.211626042092204 |
| 5+10 | 0.108665062865962 |
| 10+10 | 0.0747582951295901 |
| 20+10 | 0.0595827092127759 |12
12
12
PHA (µM)
PF (µM)
1
1
5
5
0.5
0.5
1
1
0
20
0
20
5
5
0.5
0.5
0
20
0
20
10
10
10
10
1
1
5
5
0.5
0.5
1
1
0
20
0
20
5
5
0.5
0.5
0
20
0
20
10
10
10
10
TRAIL (ng/ml)
2
10
TRAIL (ng/ml)
2
10
0
5
0
5
d
### Chart: PHA + TRAIL in HT1080
| Category | PHA + TRAIL |
|---|---|
| 0+0 | 1.0 |
| 0.5+0 | 1.003160195789572 |
| 1+0 | 1.036128403744713 |
| 5+0 | 0.890212897400561 |
| 10+0 | 0.465154683267595 |
| 20+0 | 0.118055885567647 |
| 0+2 | 0.89652140854441 |
| 0.5+2 | 0.840980373520887 |
| 1+2 | 0.852742004467044 |
| 5+2 | 0.774663783681034 |
| 10+2 | 0.136969538563893 |
| 20+2 | 0.0988689825595211 |
| 0+5 | 0.866998526826023 |
| 0.5+5 | 0.814688970203869 |
| 1+5 | 0.844829634557812 |
| 5+5 | 0.45614931331084 |
| 10+5 | 0.133417288409448 |
| 20+5 | 0.0818324383405408 |
| 0+10 | 0.84260799315687 |
| 0.5+10 | 0.832093807917123 |
| 1+10 | 0.754835337166755 |
| 5+10 | 0.374471320629188 |
| 10+10 | 0.148065865133299 |
| 20+10 | 0.0862757211424227 |
### Chart: PF + TRAIL in HT1080
| Category | PF + TRAIL |
|---|---|
| 0+0 | 1.0 |
| 0.5+0 | 0.963668113165748 |
| 1+0 | 0.914500175925563 |
| 5+0 | 0.71722897689511 |
| 10+0 | 0.501726676831254 |
| 20+0 | 0.266312209234138 |
| 0+2 | 0.896138759659616 |
| 0.5+2 | 0.891095560160027 |
| 1+2 | 0.912154501739708 |
| 5+2 | 0.690918331443763 |
| 10+2 | 0.355017788029243 |
| 20+2 | 0.183431721333907 |
| 0+5 | 0.924573543401488 |
| 0.5+5 | 1.01634153016146 |
| 1+5 | 0.908662053507434 |
| 5+5 | 0.47215815056622 |
| 10+5 | 0.319402634974003 |
| 20+5 | 0.124477110129403 |
| 0+10 | 0.930164066877777 |
| 0.5+10 | 0.927127721959419 |
| 1+10 | 1.013318216766358 |
| 5+10 | 0.443228168940668 |
| 10+10 | 0.317356685823007 |
| 20+10 | 0.154801464743214 |12
12
12
PHA (µM)
1
1
5
5
0.5
0.5
1
1
0
20
0
20
5
5
0.5
0.5
PF (µM)
0
20
0
20
10
10
10
10
1
1
5
5
0.5
0.5
1
1
0
20
0
20
5
5
0.5
0.5
0
20
0
20
10
10
10
10
TRAIL (ng/ml)
2
10
TRAIL (ng/ml)
2
10
0
5
0
5
