# Supplementary material for: Combination therapy with c-met inhibitor and TRAIL enhances apoptosis in dedifferentiated liposarcoma patient-derived cells
Source: BMC Cancer. 2019 May 24;19:496. doi: 10.1186/s12885-019-5713-2 (PMC6534902; doi:10.1186/s12885-019-5713-2)
Supplement: Supplementary file 4 — Figure S2. Expression levels of death receptors and c-Met receptor expression in STS cell lines. TRAIL receptors, decoy receptor 1 (DcR1), decoy receptor 2, (DcR2), DR4, DR5, and c-Met expression levels in MFHino (a) SW872 (b), and HT1080 (c) cells, as analyzed by flow cytometry (isotype: shaded gray histogram; each receptors: bold black open histogram). (PPTX 129 kb) [file 12885_2019_5713_MOESM4_ESM.pptx]

## Slide 1
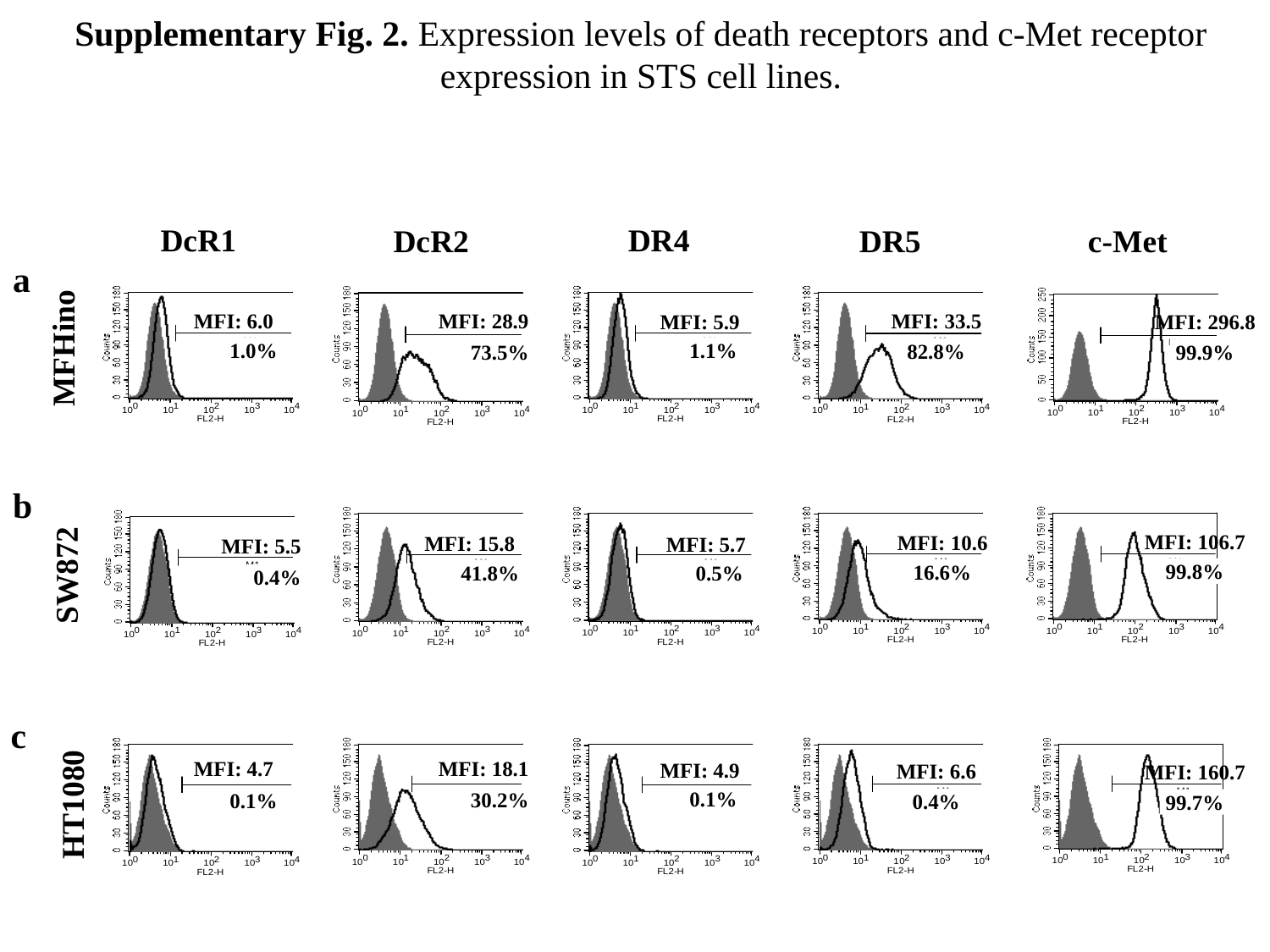

Supplementary Fig. 2. Expression levels of death receptors and c-Met receptor expression in STS cell lines.
DcR1
DR4
c-Met
DcR2
DR5
a
MFI: 28.9
MFI: 6.0
MFI: 33.5
MFI: 296.8
MFI: 5.9
MFHino
1.0%
1.1%
82.8%
73.5%
99.9%
b
MFI: 106.7
MFI: 10.6
MFI: 15.8
MFI: 5.7
MFI: 5.5
SW872
99.8%
16.6%
41.8%
0.5%
0.4%
c
MFI: 18.1
MFI: 4.7
MFI: 4.9
MFI: 6.6
MFI: 160.7
HT1080
0.1%
30.2%
0.1%
0.4%
99.7%
