# Supplementary material for: Combination therapy with c-met inhibitor and TRAIL enhances apoptosis in dedifferentiated liposarcoma patient-derived cells
Source: BMC Cancer. 2019 May 24;19:496. doi: 10.1186/s12885-019-5713-2 (PMC6534902; doi:10.1186/s12885-019-5713-2)
Supplement: Supplementary file 5 — Figure S3. c-Met inhibitor, PF and TRAIL treatment induced apoptosis in DDLPS cell lines. Apoptosis were induced through treatment with the c-Met inhibitor PF and rhTRAIL in liposarcoma cell lines. FACS plot showing apoptosis in ADMSCs (A) and SW872 cells (B) following 48 h of incubation with rhTRAIL (0, 2, 5 ng/ml) and PF (0, 5 μM) using annexin V and 7AAD. (PPTX 343 kb) [file 12885_2019_5713_MOESM5_ESM.pptx]

## Slide 1
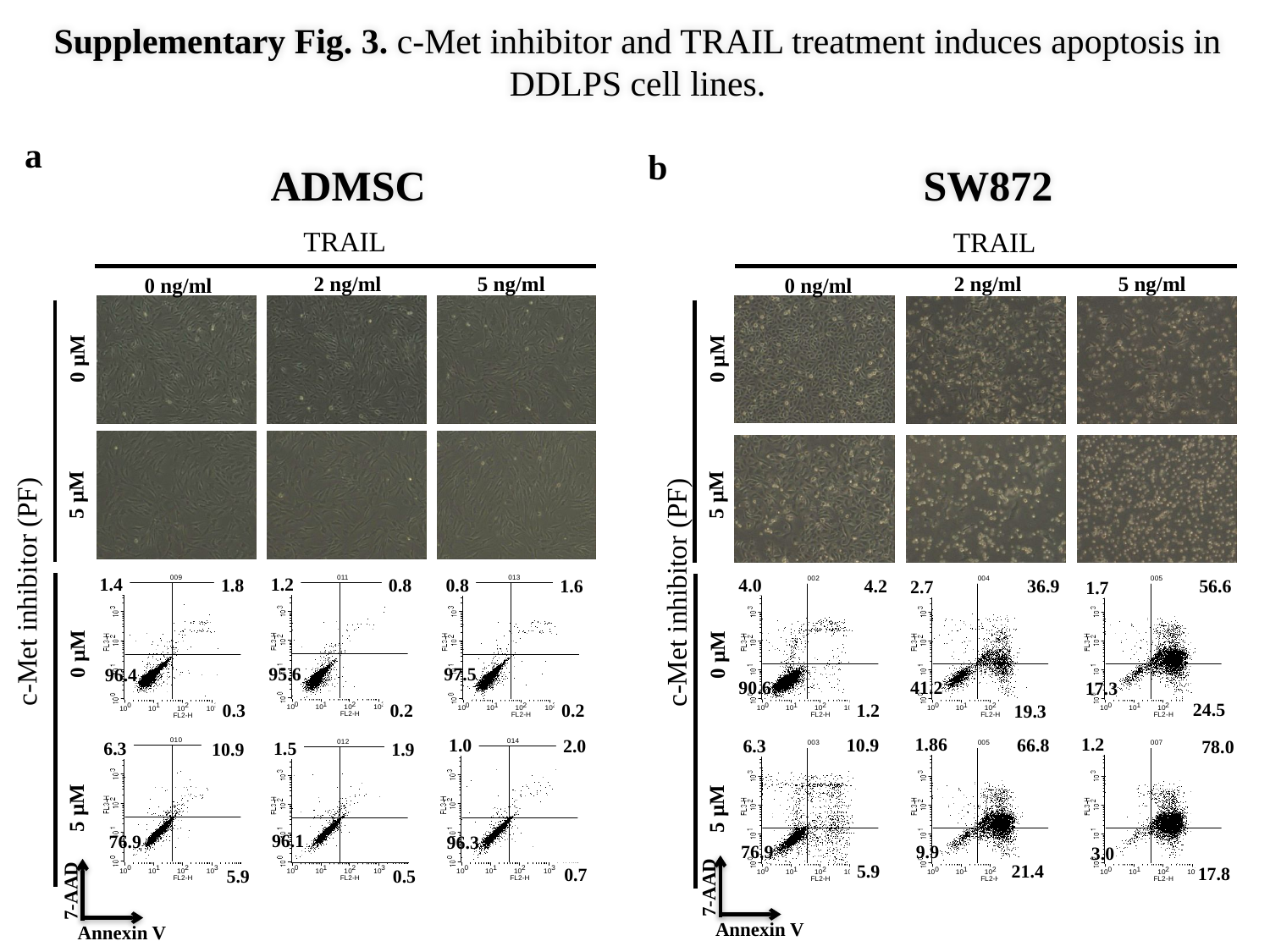

Supplementary Fig. 3. c-Met inhibitor and TRAIL treatment induces apoptosis in DDLPS cell lines.
ADMSC
SW872
a
b
TRAIL
TRAIL
2 ng/ml
5 ng/ml
2 ng/ml
5 ng/ml
0 ng/ml
0 ng/ml
0 µM
0 µM
5 µM
5 µM
c-Met inhibitor (PF)
c-Met inhibitor (PF)
1.4
1.2
4.0
0.8
1.8
0.8
4.2
1.6
36.9
56.6
2.7
1.7
0 µM
0 µM
95.6
97.5
96.4
90.6
41.2
17.3
24.5
0.3
0.2
0.2
1.2
19.3
1.86
1.2
10.9
66.8
1.0
2.0
6.3
78.0
6.3
1.5
10.9
1.9
5 µM
5 µM
96.1
76.9
96.3
76.9
9.9
3.0
5.9
21.4
17.8
0.7
5.9
0.5
7-AAD
7-AAD
Annexin V
Annexin V
