# Supplementary material for: Combination therapy with c-met inhibitor and TRAIL enhances apoptosis in dedifferentiated liposarcoma patient-derived cells
Source: BMC Cancer. 2019 May 24;19:496. doi: 10.1186/s12885-019-5713-2 (PMC6534902; doi:10.1186/s12885-019-5713-2)
Supplement: Supplementary file 6 — Figure S4. Efficacy of tumor cell suppression through combined treatment with the c-Met inhibitor, PF and rhTRAIL in DDLPS PDCs. Human liposarcoma cells were treated with PF (5 μM) and rhTRAIL (5 ng/ml) for 48 h. Cell viability was analyzed by CCK8: (a) rhTRAIL only (5 ng/ml) (b) PF only (5 μM) (c) combination treatment with PF (5 μM) and rhTRAIL (5 ng/ml). (PPTX 102 kb) [file 12885_2019_5713_MOESM6_ESM.pptx]

## Slide 1
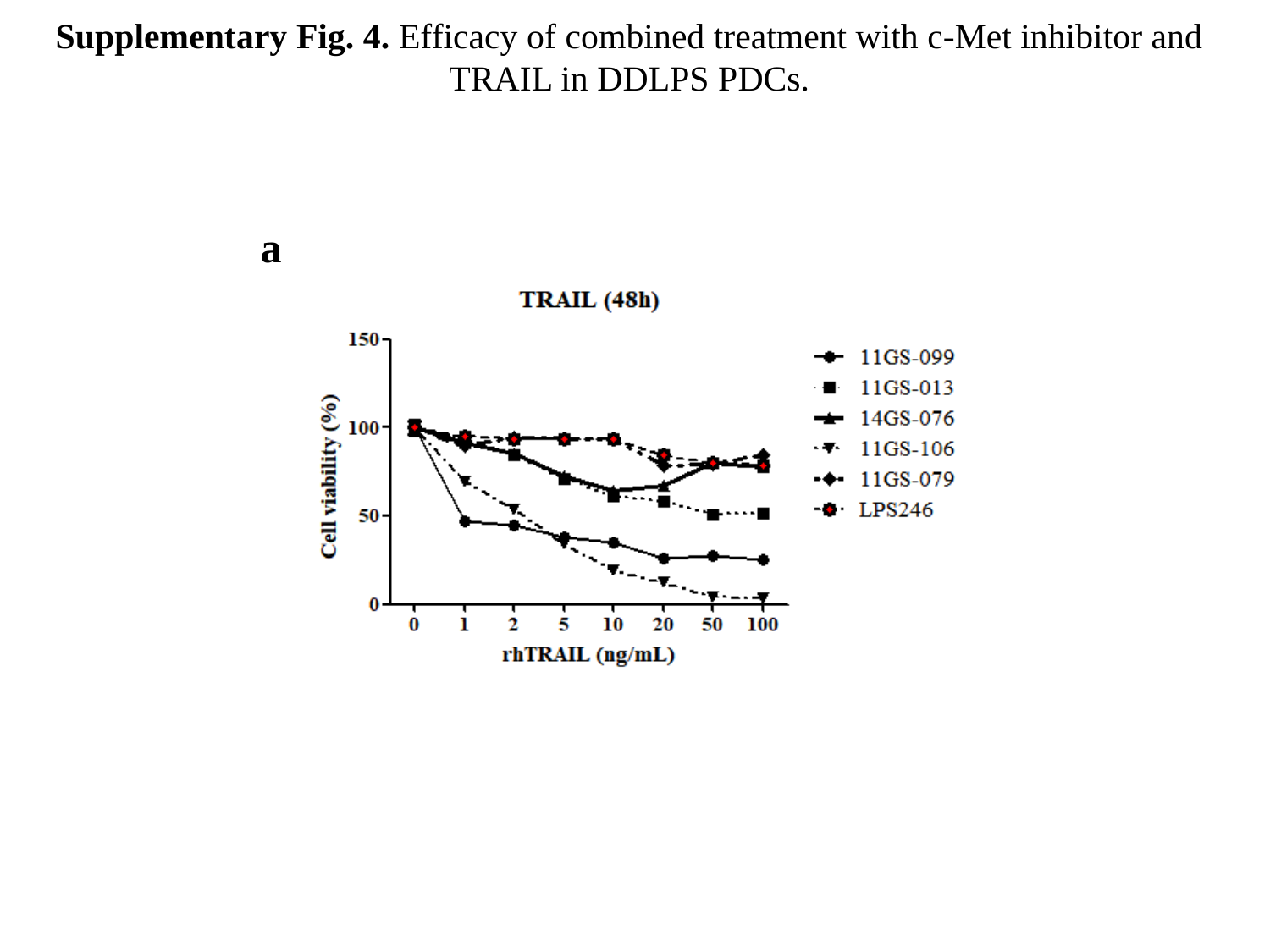

Supplementary Fig. 4. Efficacy of combined treatment with c-Met inhibitor and TRAIL in DDLPS PDCs.
a

## Slide 2
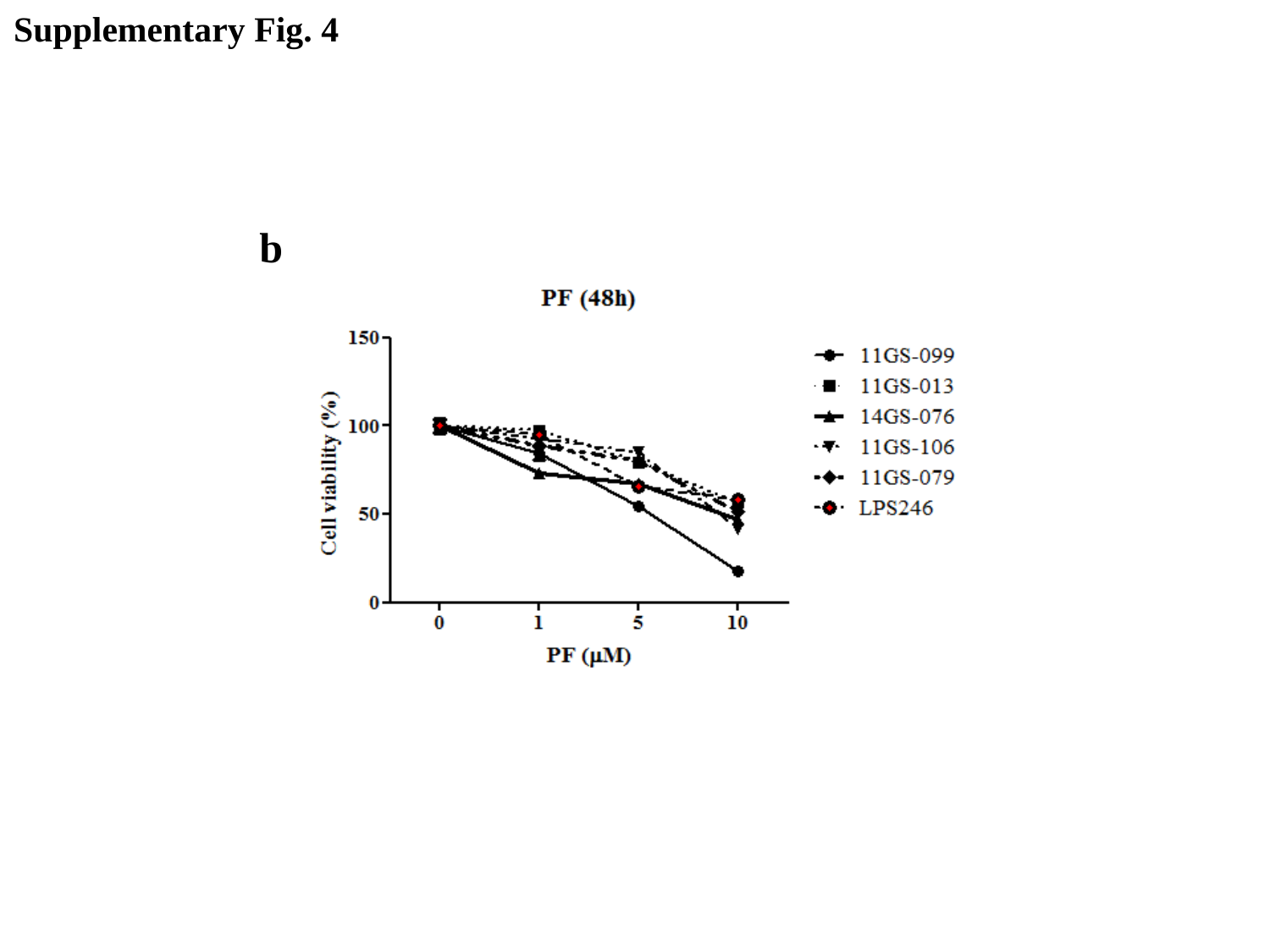

Supplementary Fig. 4
b

## Slide 3
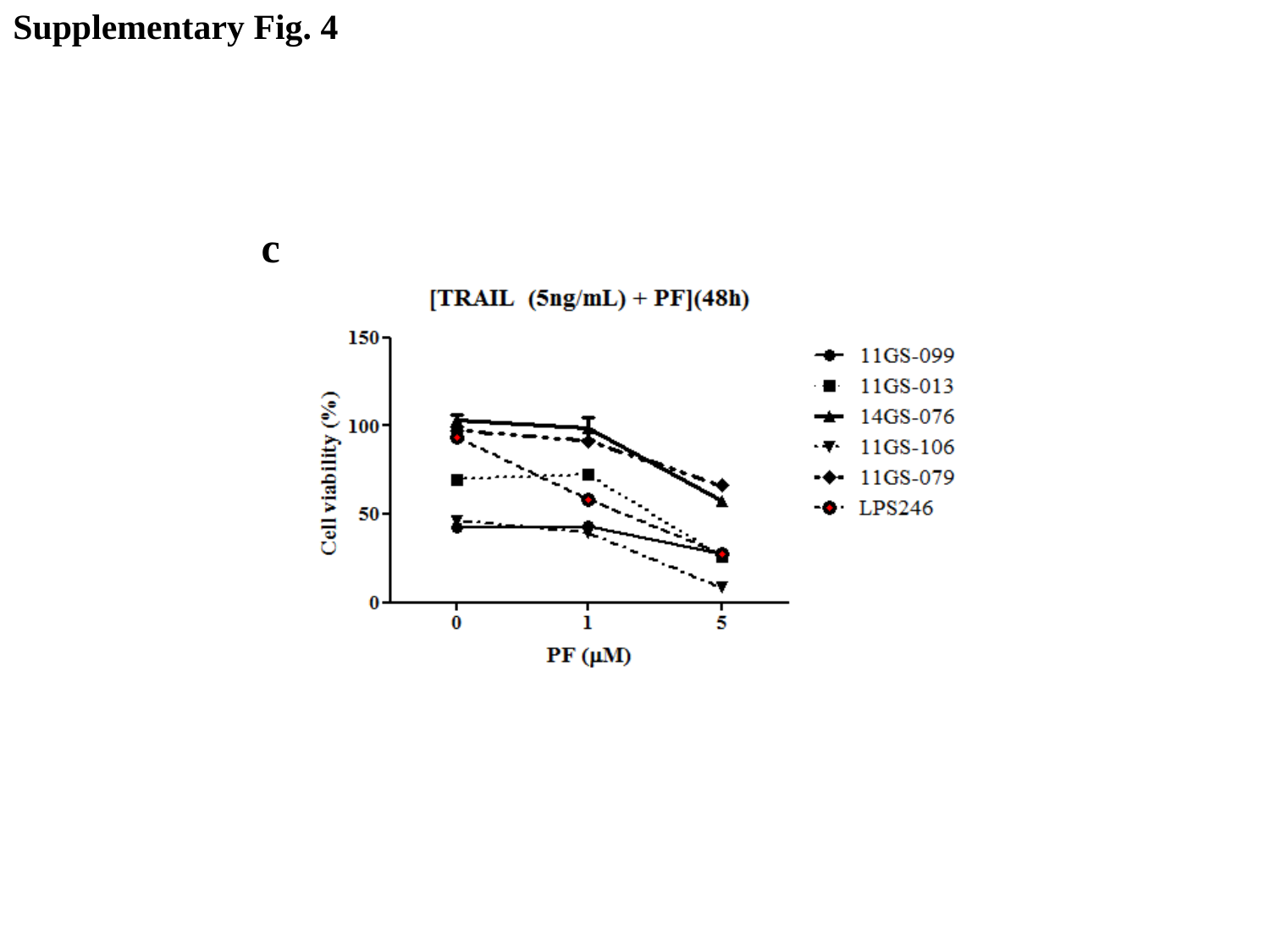

Supplementary Fig. 4
c
