# Supplementary material for: Combination therapy with c-met inhibitor and TRAIL enhances apoptosis in dedifferentiated liposarcoma patient-derived cells
Source: BMC Cancer. 2019 May 24;19:496. doi: 10.1186/s12885-019-5713-2 (PMC6534902; doi:10.1186/s12885-019-5713-2)
Supplement: Supplementary file 7 — Figure S5. Cell death was induced by PF and/ or rhTRAIL treatment. Representative Western blots of caspase 3, caspase 7, caspase 8, Bcl2, PARP, DR4 and DR5 were shown. Membranes were re-probed for ACTB expression to show that similar amounts of protein were loaded in each lane for LPS246 cells (a) and 11GS079 PDC (b). (1) primary treatment, (2) secondary treatment. (PPTX 307 kb) [file 12885_2019_5713_MOESM7_ESM.pptx]

## Slide 1
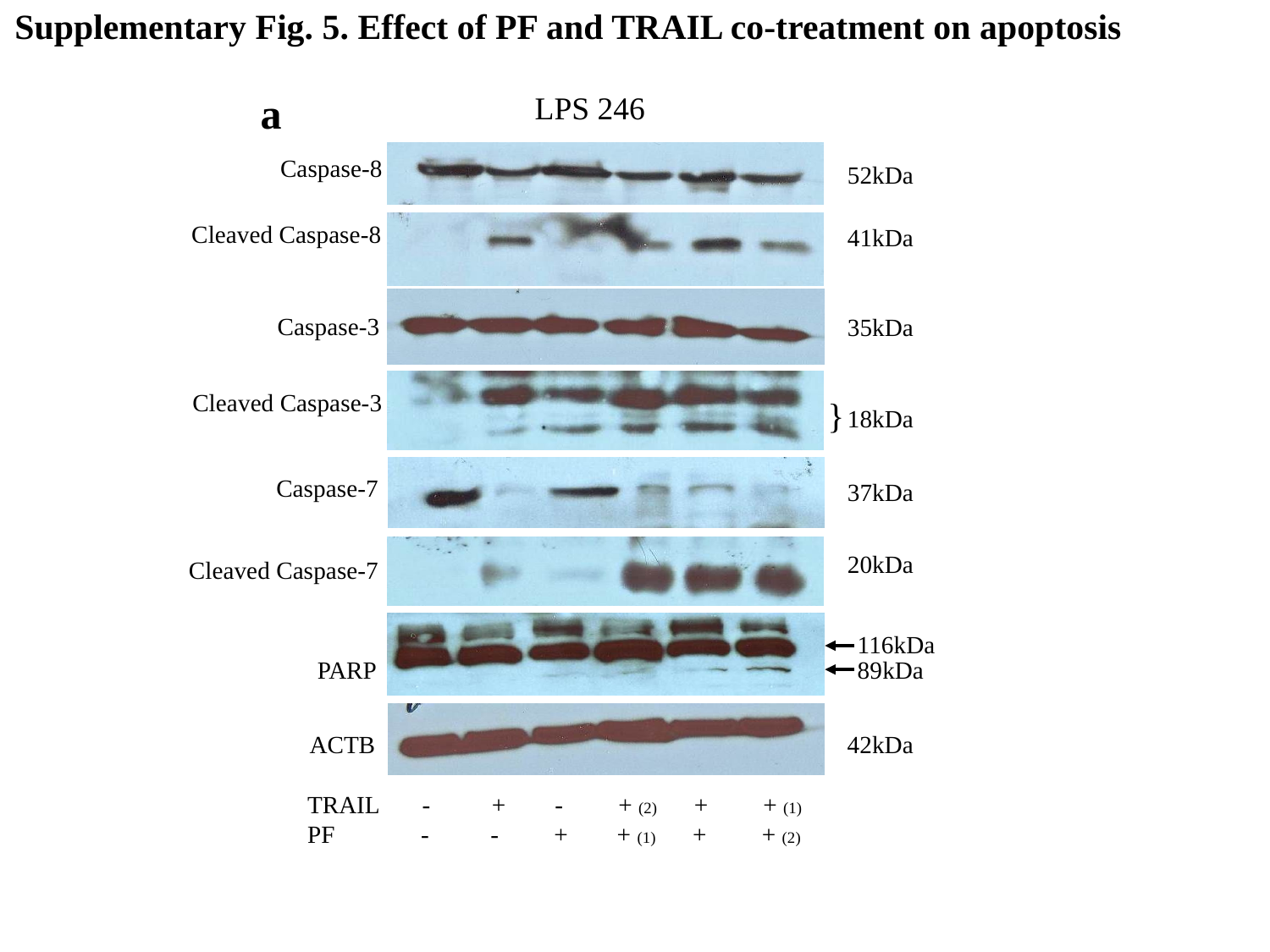

Supplementary Fig. 5. Effect of PF and TRAIL co-treatment on apoptosis
a
LPS 246
Caspase-8
52kDa
Cleaved Caspase-8
41kDa
Caspase-3
35kDa
Cleaved Caspase-3
}
18kDa
Caspase-7
37kDa
20kDa
Cleaved Caspase-7
116kDa
PARP
89kDa
42kDa
ACTB
TRAIL - + - + (2) + + (1)
PF - - + + (1) + + (2)

## Slide 2
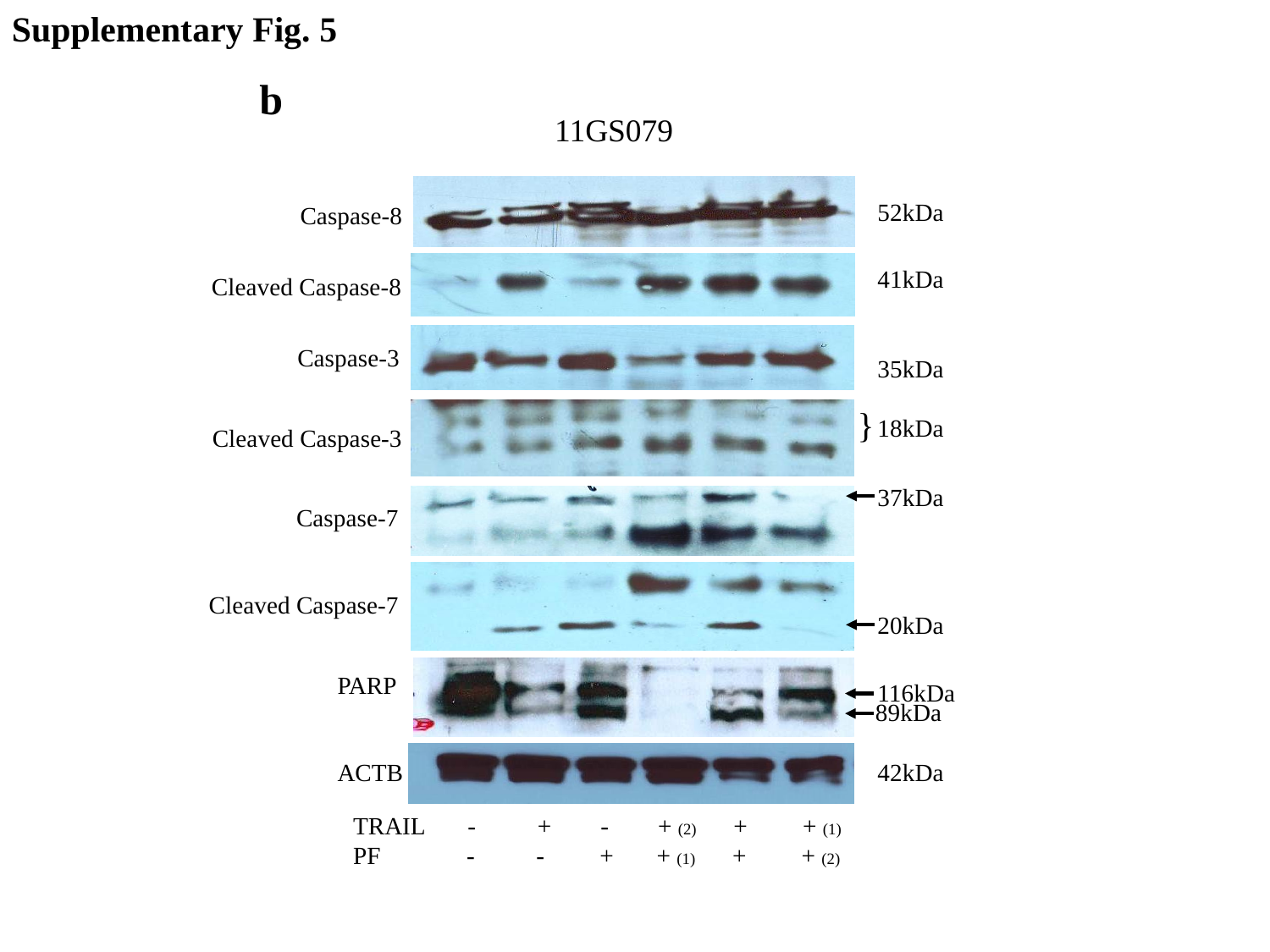

Supplementary Fig. 5
b
11GS079
52kDa
Caspase-8
41kDa
Cleaved Caspase-8
Caspase-3
35kDa
}
18kDa
Cleaved Caspase-3
37kDa
Caspase-7
Cleaved Caspase-7
20kDa
PARP
116kDa
89kDa
42kDa
ACTB
TRAIL - + - + (2) + + (1)
PF - - + + (1) + + (2)
