# Supplementary figures and images for: Combination therapy with c-met inhibitor and TRAIL enhances apoptosis in dedifferentiated liposarcoma patient-derived cells
Source: BMC Cancer. 2019 May 24;19:496. doi: 10.1186/s12885-019-5713-2 (PMC6534902; doi:10.1186/s12885-019-5713-2)

## Slide 1
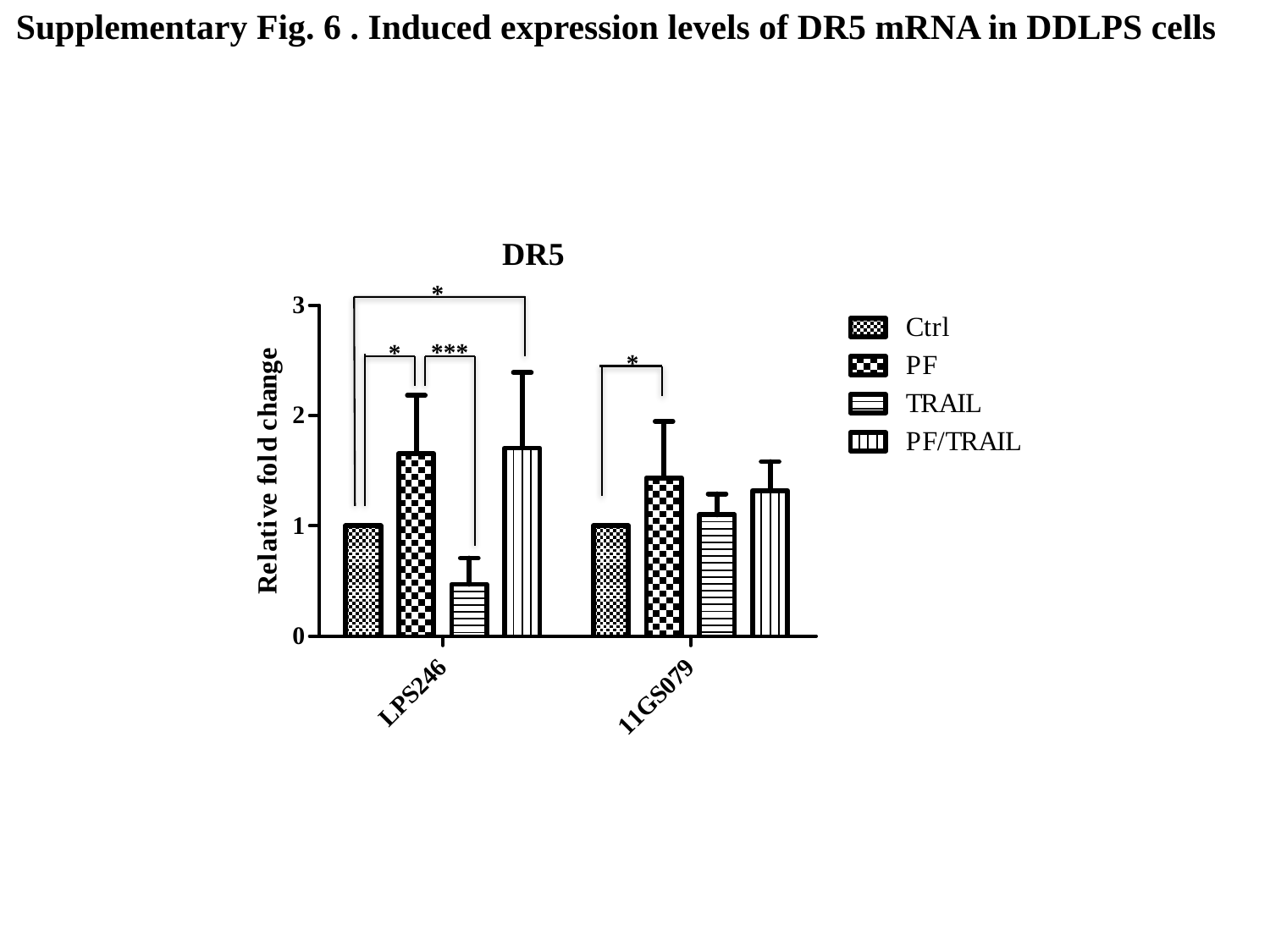

Supplementary Fig. 6 . Induced expression levels of DR5 mRNA in DDLPS cells
*
***
*
*

Supplement: Supplementary file 8 — Figure S6. Induced expression levels of DR5 mRNA in DDLPS cells by PF and/ or rhTRAIL treatment. DR5 mRNA expression levels were detected in DDLPS cells after treatment with PF and/ or rhTRAIL. LPS246 and 11GS079 cells were treated with DMSO (as control), PF (5 μM), rhTRAIL (5 ng/mL) and PF (5 μM) with rhTRAIL (5 ng/mL) simultaneously for 48 h. RNA samples were isolated and subjected to real-time PCR analysis. Data were normalized GAPDH level and presented as fold changes in fluorescence density compared to that of the control group. Data are shown as the mean ± SD. *, P < 0.05; ***, P < 0.001 versus control. (PPTX 68 kb) [file 12885_2019_5713_MOESM8_ESM.pptx]
