# Supplementary material for: Combination therapy with c-met inhibitor and TRAIL enhances apoptosis in dedifferentiated liposarcoma patient-derived cells
Source: BMC Cancer. 2019 May 24;19:496. doi: 10.1186/s12885-019-5713-2 (PMC6534902; doi:10.1186/s12885-019-5713-2)
Supplement: Supplementary file 9 — Figure S7. Death receptor was up-regulated by c-Met inhibitor, PF. Representative Western blot results of Bcl2, DR4 and DR5 were shown. Membranes were re-probed for ACTB expression to show that similar amounts of protein were loaded in each lane in LPS246 cells (a) and 11GS079 PDCs (b). (1) primary treatment, (2) secondary treatment. (PPTX 156 kb) [file 12885_2019_5713_MOESM9_ESM.pptx]

## Slide 1
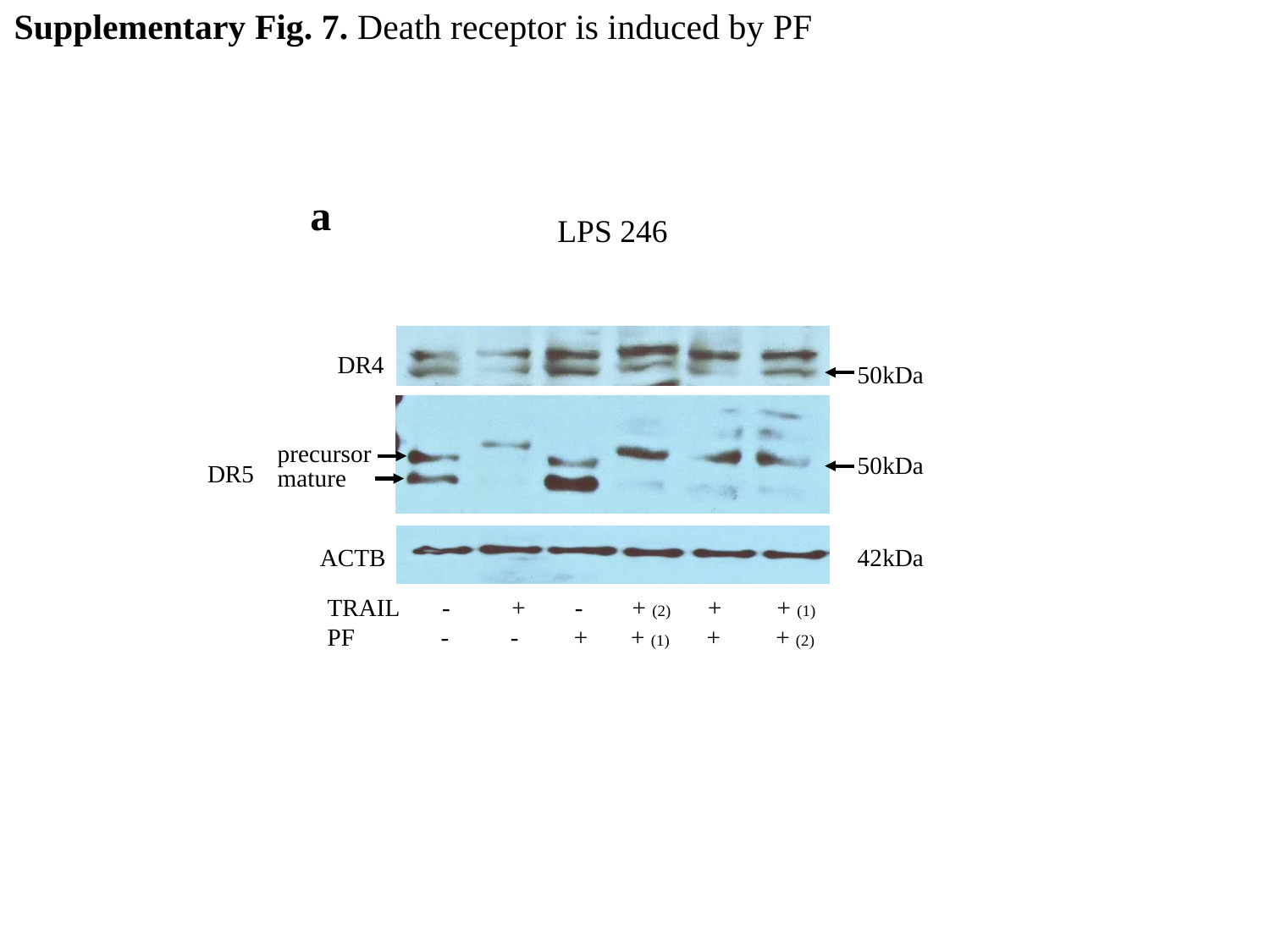

Supplementary Fig. 7. Death receptor is induced by PF
a
LPS 246
DR4
50kDa
precursor
50kDa
DR5
mature
42kDa
ACTB
TRAIL - + - + (2) + + (1)
PF - - + + (1) + + (2)

## Slide 2
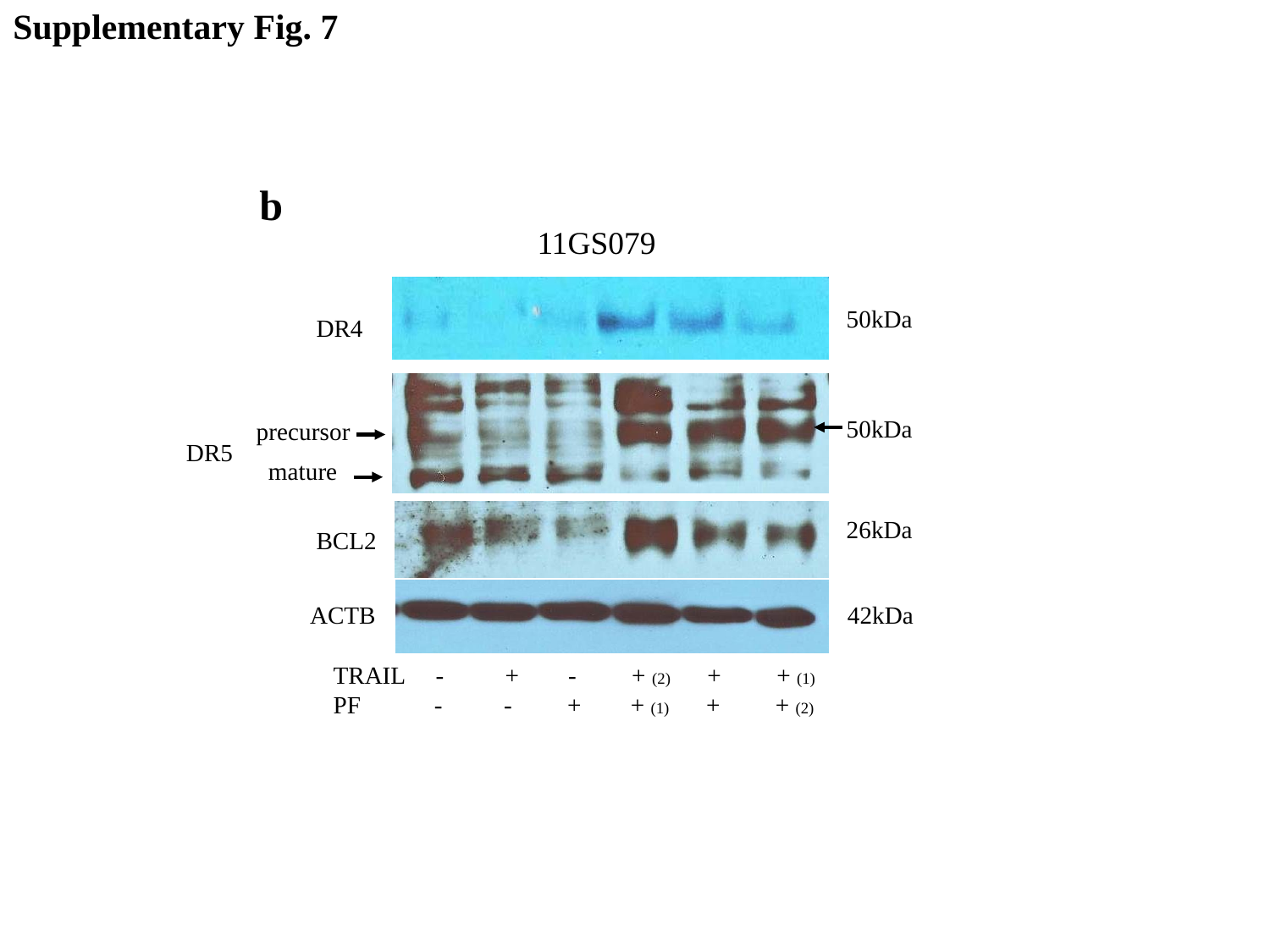

Supplementary Fig. 7
b
11GS079
50kDa
DR4
50kDa
precursor
DR5
mature
26kDa
BCL2
42kDa
ACTB
TRAIL - + - + (2) + + (1)
PF - - + + (1) + + (2)
