# Supplementary material for: Combination therapy with c-met inhibitor and TRAIL enhances apoptosis in dedifferentiated liposarcoma patient-derived cells
Source: BMC Cancer. 2019 May 24;19:496. doi: 10.1186/s12885-019-5713-2 (PMC6534902; doi:10.1186/s12885-019-5713-2)
Supplement: Supplementary file 10 — Figure S8. Effect of apoptosis by combination treatment with PF and/ or rhTRAIL and combined with DR5 siRNA. To determine the direct roles of DR5 in PF-induced TRAIL sensitization, LPS224 cells were treated with DR5 siRNA, followed by co-treatment with PF (5 μM) and rhTRAIL (5 ng/ml) for 48 h. Representative Western blots of caspase-3, caspase-7 (a), and caspase-8 (b) were shown. (PPTX 275 kb) [file 12885_2019_5713_MOESM10_ESM.pptx]

## Slide 1
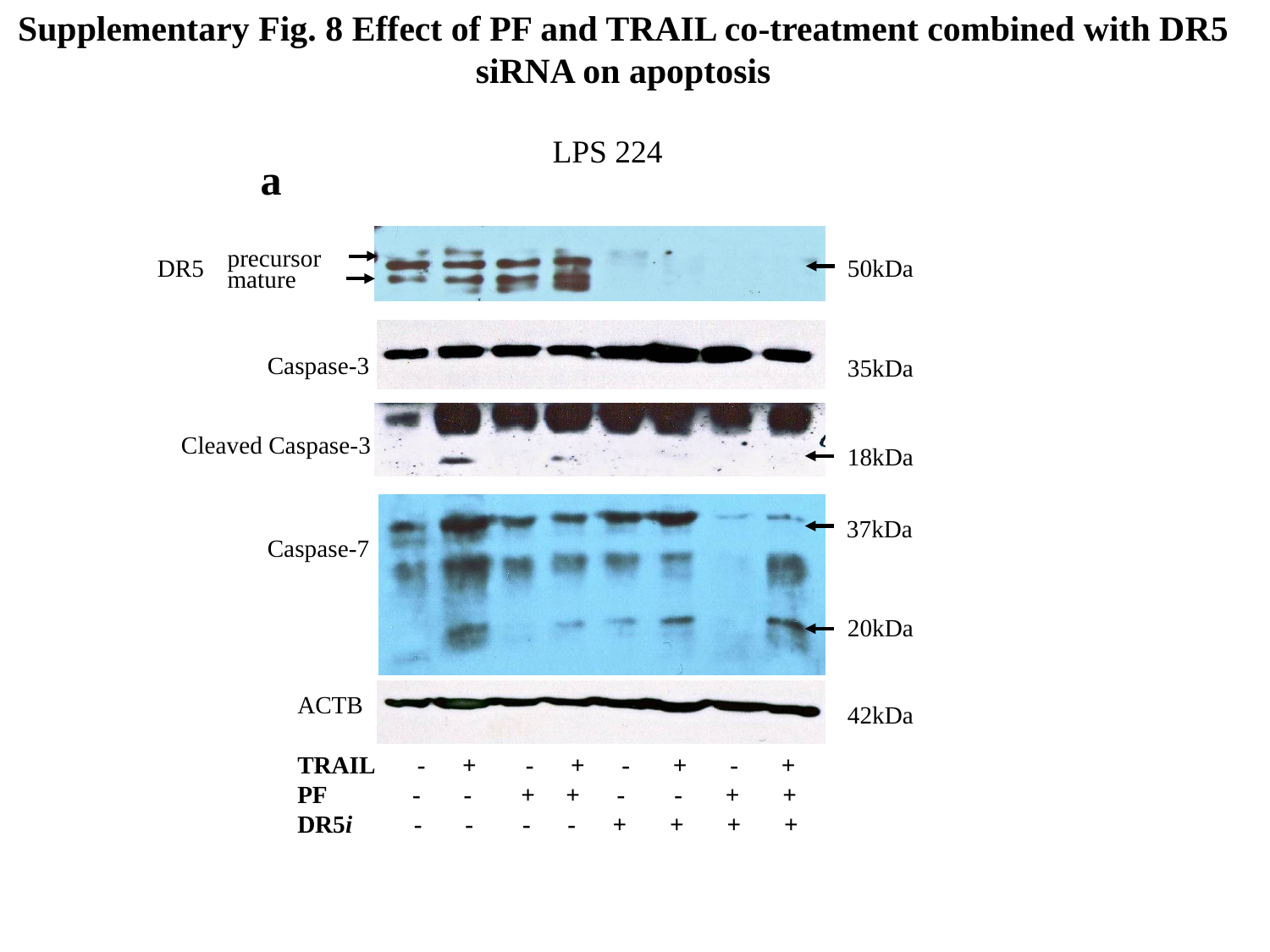

Supplementary Fig. 8 Effect of PF and TRAIL co-treatment combined with DR5 siRNA on apoptosis
LPS 224
a
precursor
DR5
50kDa
mature
Caspase-3
35kDa
Cleaved Caspase-3
18kDa
37kDa
Caspase-7
20kDa
ACTB
42kDa
TRAIL - + - + - + - +
PF - - + + - - + +
DR5i - - - - + + + +

## Slide 2
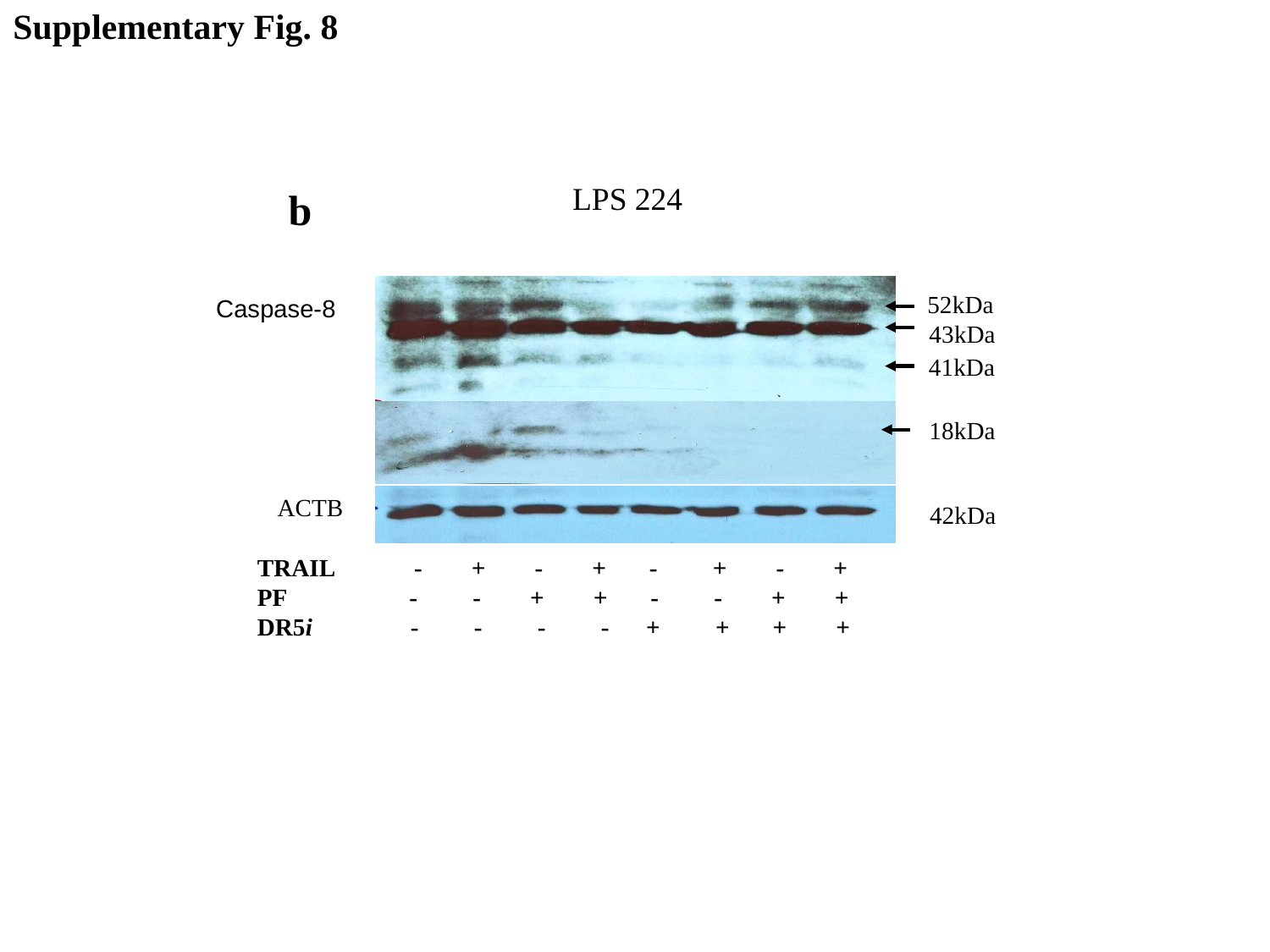

Supplementary Fig. 8
LPS 224
b
52kDa
Caspase-8
43kDa
41kDa
18kDa
ACTB
42kDa
TRAIL - + - + - + - +
PF - - + + - - + +
DR5i - - - - + + + +
