# Supplementary material for: Combination therapy with c-met inhibitor and TRAIL enhances apoptosis in dedifferentiated liposarcoma patient-derived cells
Source: BMC Cancer. 2019 May 24;19:496. doi: 10.1186/s12885-019-5713-2 (PMC6534902; doi:10.1186/s12885-019-5713-2)
Supplement: Supplementary file 11 — Figure S9. c-Met and rhTRAIL receptor expression levels in DDLPS. PDCs. c-Met inhibitor PF upregulated expression levels of c-Met in DDLPS PDCs. The expression levels of DcR1, DcR2, DR4, DR5, and c-Met were analyzed by flow cytometry after DMSO (vehicle: shaded gray histogram) and PF (5 μM: bold black open histogram) treatment for 48 h, as shown in the upper column (a). c-Met expression levels in LPS224, LPS246, 11GS-013, 11GS-079, 11GS-099, 11GS-106 and 11GS-076 cells were analyzed by flow cytometry (b). (PPTX 513 kb) [file 12885_2019_5713_MOESM11_ESM.pptx]

## Slide 1
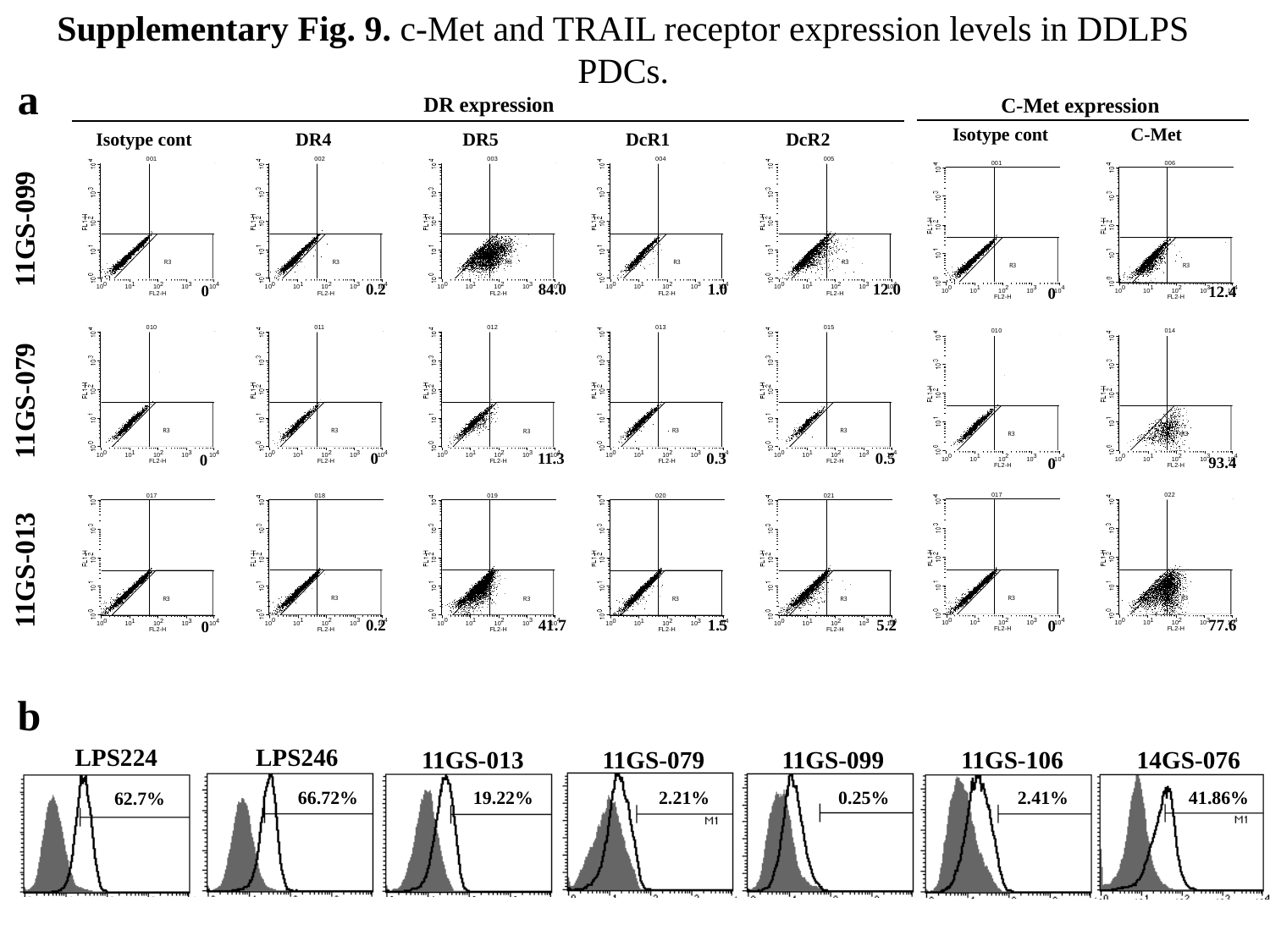

Supplementary Fig. 9. c-Met and TRAIL receptor expression levels in DDLPS PDCs.
a
DR expression
C-Met expression
Isotype cont
C-Met
Isotype cont
DR4
DR5
DcR1
DcR2
11GS-099
0.2
84.0
1.0
12.0
0
12.4
0
11GS-079
0
11.3
0.3
0.5
0
93.4
0
11GS-013
77.6
0.2
41.7
1.5
5.2
0
0
b
LPS224
LPS246
11GS-013
11GS-079
11GS-099
11GS-106
14GS-076
66.72%
19.22%
2.21%
0.25%
2.41%
41.86%
62.7%
